# Tenascin-C fibronectin D domain is involved in the fine-tuning of glial response to CNS injury *in vitro*

**DOI:** 10.3389/fcell.2022.952208

**Published:** 2022-08-26

**Authors:** Dunja Bijelić, Marija Adžić, Mina Perić, Gebhard Reiss, Milena Milošević, Pavle R. Andjus, Igor Jakovčevski

**Affiliations:** ^1^ Centre for Laser Microscopy, Institute of Physiology and Biochemistry “Ivan Djaja”, Faculty of Biology, University of Belgrade, Belgrade, Serbia; ^2^ Laboratory for Human Molecular Genetics, Institute of Molecular Genetics and Genetic Engineering, University of Belgrade, Belgrade, Serbia; ^3^ Institute for Anatomy and Clinical Morphology, University Witten / Herdecke, Witten, Germany

**Keywords:** tenascin-C, fibronectin III-like domain D, scratch wound, microglia, astrocytes, co-culture

## Abstract

Understanding processes that occur after injuries to the central nervous system is essential in order to gain insight into how the restoration of function can be improved. Extracellular glycoprotein tenascin-C (TnC) has numerous functions in wound healing process depending on the expression time, location, isoform and binding partners which makes it interesting to study in this context. We used an *in vitro* injury model, the mixed culture of cortical astrocytes and microglia, and observed that without TnC microglial cells tend to populate gap area in greater numbers and proliferate more, whereas astrocytes build up in the border region to promote faster gap closure. Alternatively spliced domain of TnC, fibronectin type III-like repeat D (FnD) strongly affected physiological properties and morphology of both astrocytes and microglia in this injury model. The rate of microglial proliferation in the injury region decreased significantly with the addition of FnD. Additionally, density of microglia also decreased, in part due to reduced proliferation, and possibly due to reduced migration and increased contact inhibition between enlarged FnD-treated cells. Overall morphology of FnD-treated microglia resembled the activated pro-inflammatory cells, and elevated expression of iNOS was in accordance with this phenotype. The effect of FnD on astrocytes was different, as it did not affect their proliferation, but stimulated migration of reactivated astrocytes into the scratched area 48 h after the lesion. Elevated expression and secretion of TNF-α and IL-1β upon FnD treatment indicated the onset of inflammation. Furthermore, on Western blots we observed increased intensity of precursor bands of β1 integrin and appearance of monomeric bands of P2Y12R after FnD treatment which substantiates and clarifies its role in cellular shape and motility changes. Our results show versatile functions of TnC and in particular FnD after injury, mostly contributing to ongoing inflammation in the injury region. Based on our findings, FnD might be instrumental in limiting immune cell infiltration, and promoting astrocyte migration within the injury region, thus influencing spaciotemporal organization of the wound and surrounding area.

## Introduction

Complete recovery from mechanical injuries of the central nervous system (CNS) is challenging goal in regenerative medicine research. Due to the death of neurons within the injury region and small capacity for regeneration of the damaged ones, the injuries always result in a partial or total loss of various sensorimotor functions (Ahuja et al., 2017). CNS injuries begin with the initial mechanical tissue damage that immediately disrupts cerebral blood flow and metabolism, followed by a secondary cascade of neuroinflammatory events (Werner and Engelhard, 2007). A multi-stage process of glial scar creation begins to limit and close the wound, but previous tissue structure and functions are never completely restored. Thus, all regenerative interventions focus on understanding and modulating the events in the secondary injury in attempt to create ideal environment for nerve recovery and guidance. Within the first few days after injury, numerous damage-associated molecular patterns (DAMPs) released from damaged cells and blood induce reactivation and proliferation of glial and stromal cells, increased deposition of extracellular matrix and recruitment of circulating innate and adaptive immune cells (Bradbury and Burnside, 2019). The injury center is invaded by stromal fibroblasts and inflammatory cells of the immune system, while reactive hypertrophied astrocytes form a barrier that borders healthy tissue (Beck et al., 2010; Susarla et al., 2014; Karve et al., 2016; Zhou et al., 2020; Escartin et al., 2021). From a mixed pro-inflammatory/protective phenotype, macrophages/microglia become predominantly pro-inflammatory and spread inflammation further into the tissue by engaging adaptive immune cells (Orihuela et al., 2016), and affecting astrocyte phenotype in inflammation (Liddelow et al., 2017; Bellver-Landete et al., 2019).

One of the damage-associated molecules is the extracellular glycoprotein tenascin-C (TnC). This protein is present in the extracellular matrix of vertebrates during embryonic development (Bartsch et al., 1992). In adult tissues, upregulation of TnC has been shown upon tissue injury and cellular stress in inflammation and tissue remodelling (Midwood and Orend, 2009; Tucker and Chiquet-Ehrismann, 2009; Jakovcevski et al., 2013; Marzeda and Midwood, 2018). TnC expression is regulated by a number of cytokines, transcription factors and intracellular regulators reviewed in detail elsewhere (Midwood et al., 2016; Okada and Suzuki, 2021). In the CNS, after injury, it is intensively synthesized and secreted into the extracellular matrix by astrocytes (Laywell et al., 1992; Wiese et al., 2012). Starting from the N-terminus, TnC monomer is made up of tenascin assembly domain (TA), followed by 14.5 epidermal growth factor-like repeats (EGFL), then up to 17 fibronectin type III-like repeats (FnIII) and fibrinogen-like globe (FG) at the C-terminus (Nies et al., 1991; Giblin and Midwood, 2015). Through the TA domain, six monomers are assembled into the hexabrachion structure (Taylor et al., 1989; Erickson, 1993). Within FnIII repeats, in addition to the constitutively expressed domains (Fn1-8), there are also nine alternatively spliced ​​ones (FnA1/2/3/4, B, AD2, AD1, C, D) situated between Fn5 and 6 (Jones and Jones, 2000). By alternative splicing the expression of various isoforms with different number of domains is possible, resulting in a plethora of tenascin C functions (Gulcher et al., 1989; Nies et al., 1991; Jones and Jones, 2000; Giblin and Midwood, 2015). TnC isoforms with lower molecular mass (<200 kDa) do not contain alternatively spliced domains and are usually present in physiologically normal tissues, whereas isoforms with larger molecular mass (>200 kDa) contain at least one alternatively spliced repeat and are upregulated in tumors and after injuries (Giblin and Midwood, 2015; Okada and Suzuki, 2021).

TnC interacts with over 25 molecules that are classified into cell receptors, extracellular matrix components, pathogenic components and soluble factors and thus modulates the processes of cell migration, adhesion, proliferation, survival, neurite outgrowth, extracellular matrix reorganization, synthesis of proteases and cytokines (Giblin and Midwood, 2015). Some of the main TnC roles were shown to be adhesion-modulation by binding to fibronectin, alterations in cell spreading and signalling through fibronectin and integrins, modulation of inflammatory signalling pathways through Toll-like receptor 4 (TLR4) (Midwood et al., 2016). The role of TnC has been extensively investigated in cell adhesion and migration of both neuronal and non-neuronal cells as well as in their interaction during CNS development and remodelling. TnC has boundary-like tissue distribution that matches the areas of intense neuronal migration, guidance, neurite outgrowth and reveals its role ultimately in segregation of neurons and their fibre tracts into functional units (Bartsch et al., 1992; Faissner and Steindler, 1995; Scholze et al., 1996; Faissner, 1997).

Although *in vitro* injury models lack some of the components present *in vivo* (Yoo et al., 2016), they are often a method of choice for the study of acute or subacute pathophysiology after a trauma stimulus, as they provide precise control on the extracellular environment and easy and repeatable access to the cells (Slovinska et al., 2016; Goshi et al., 2020).

In our previous study, the effects of various TnC domains were tested in *in vitro* injury model performed in wild-type and TnC-deficient cortical astrocytes. We observed a delay in gap closure caused by TnC fragments, in part due to strongly reduced cell proliferation and this effect was the most pronounced when we applied FnD fragment (Bijelić et al., 2021). This is coupled with the beneficial effect of FnD fragment on regeneration and motor recovery after spinal cord injury published earlier (Chen et al., 2010). Given the importance of microglia in glial scar formation we further wanted to examine the effects of FnD on both, astrocytes and microglia in mixed cortical cultures that more closely mimics the interaction of glial cells in tissue. Our new results show that in mixed cell cultures of astrocytes and microglia, addition of FnD decreased the proliferation and density of microglia, but promoted migration of astrocytes into the gap area. Microglia and astrocytes became reactivated, as confirmed by changed morphology, upregulated production of pro-inflammatory mediators and shifts in the expression levels of isoforms of receptors involved in cell morphology and motility. These findings indicate that, as more cell types are included in the model system, more roles of tenascin-C and its domain FnD emerge.

## Materials and methods

### Animals

Local colony of wild-type C57BL/6 (TnC^+/+^) mice and constitutively tenascin-C deficient (TnC^−/−^) mice were housed in the Animal Facility of the Faculty of Biology, University of Belgrade under standard conditions: 21 ± 1°C, 50% humidity, 12:12 h light/dark cycle, water and food *ad libitum*. TnC^−/−^ mice were derived from the original colony (Evers et al., 2002) and were inbred on the C57BL/6 background for more than 10 generations. All experiments on cell cultures isolated from 0 to 3 days old pups derived from abovementioned colonies were approved by the Ethics Committee of the Faculty of Biology, University of Belgrade and are in accordance with the NIH Guide for Care and Use of Laboratory Animals (1985) and the European Communities Council Directive (86/609/EEC).

### Cortical glial cell culture

Primary astrocyte and microglia mixed cell cultures were prepared for each genotype from cortices of 0–3 days old mice of both sexes. Cortices were isolated in ice-cold phosphate buffered saline (PBS), and tissue was enzymatically dissociated with 1 mg/ml of freshy dissolved Trypsin in PBS for 10 min at 37°C. To inactivate trypsin, four times greater amount of growth medium (Dulbecco’s modified Eagle medium, DMEM, supplemented with 10% FBS, D-glucose to a final concentration of 25 mmol/L, 100 IU/ml penicillin, and 100 μg/ml streptomycin, all from Thermo Fisher Scientific, United States-Gibco) was added to the mixture, and cell suspension was centrifuged for 8 min at 500 × g. Cells were then resuspended in supplemented DMEM, passed successively 3–5 times through 21G (ø 0.8 mm) and 23G (ø 0.6 mm) needles and centrifuged again for 8 min at 500 × g. Resuspended cells were again passed through 23G needle and seeded as per experimental requirements: in 6-well plate for Western blot and rtPCR, and on PLO coated glass coverslips (ø 15 mm, PLO - Sigma-Aldrich, St. Louis, Missouri, United States) for immunofluorescence labelling. Cultures were maintained in a humidified atmosphere of 5% CO_2_/95% air at 37°C. The culture medium was replaced 24 h after the seeding and then only half of medium was changed every 2–3 days. These mixed astrocyte and microglia cultures were grown 12–14 days *in vitro* (DIV) in total. Three cell cultures per genotype were prepared for each experiment. In these mixed cell cultures of astrocytes and microglia, on average, microglia accounted for 20% of total cells in wild-type (TnC^+/+^) cultures, and 16% in TnC^−/−^ cultures.

### Scratch wound assay and treatments

In this study**,** Scratch wound assay (SW) was performed in all experimental conditions (except in no SW) using sterile 200 μl pipette tip. This was followed by immediate or delayed addition of alternatively spliced domain FnD of protein TnC (10 μg/ml in growth medium). Recombinantly expressed fibronectin type III-like repeat D was generated as described by Dörries et al. (Dorries et al., 1996).

Experimental conditions were as follows:

• Control groups in which only SW was performed—**24** **h SW** and **48** **h SW** (immunolabeling, WB and ELISA); **6** **h SW** and **30** **h SW** (RT PCR).
• Treated groups with immediate addition of FnD upon SW—**24** **h SW + FnD** and **48** **h SW + FnD** (immunolabeling, WB and ELISA); **6** **h SW + FnD** and **30** **h SW + FnD** (RT PCR).
• Treated groups with delayed addition of FnD upon SW—**48** **h SW: 24** **h + FnD** (immunolabeling, WB and ELISA); **30** **h SW: 24** **h + FnD** (RT PCR).
• Additional groups without SW: **24** **h no SW** and **48** **h no SW** (WB, ELISA); **6** **h no SW** and **30** **h no SW** (RT PCR).

### Immunocytochemistry

For immunocytochemistry experiments cells were fixed 24 or 48 h after the SW and treatment in 4% formaldehyde solution (PFA), 20 min at RT. If the antibody for the target protein was against extracellular protein domain, the cultures were firstly blocked (5% BSA in PBS) for 1 h, at RT, then incubated with selected antibody of interest, followed by appropriate secondary fluorophore-tagged antibody. List of all used primary and secondary antibodies and their concentrations are given in Table 1. Subsequently, the cells were permeabilized (0.05% Triton in PBS) for 15 min, at RT, for further protein targets fluorescence labelling. All primary antibodies were incubated overnight, at +4°C, while the fluorescent secondary antibodies were incubated for 2 h, at RT. Cell nuclei were stained with Hoechst (2 ug/ml final dilution, for 15 min, at RT, Cat. no. 62249 Thermo Fisher Scientific, United States). Glass coverslips were mounted on microscope slides with MOWIOL solution (Sigma-Aldrich, St. Louis, Missouri, United States). The omission of the primary antibodies resulted in the absence of any specific immunoreactivity.

**TABLE 1:** List of primary and secondary antibodies used.

Target	Host/clonality	Method/dilution	Cat.no./RRID
GFAP	Mouse	IF/1:200	MAB360Milipore/AB_11,212,597
OX-42/CD11b	rat/monocl	IF/1:300	Serotec, UK(BioRad) MCA711/AB_321,292
Ki67	rabbit/polycl	IF/1:500	Abcam ab15580/AB_443209
iNOS	rabbit/polycl	IF/1:300	Abcam ab15323/AB_301857
β1-intgerin	rabbit/polycl	WB/1:1000	SynapticSystems 240–003/AB_11043176
P2Y12	rabbit/polycl	WB/1:200	Alomone APR-012/AB_2040074
anti-mouse AF555	Donkey	IF/1:200	Invitrogen A-31570/AB_2536180
anti-rat AF488	Donkey	IF/1:200	Invitrogen A-21208/AB_141709
anti-rabbit AF647	Donkey	IF/1:200	Invitrogen A-31573/AB_2536183
anti-rabbit HRP	Goat	WB/1:2000	Abcam ab6721/AB_955447
β -actin HRP	mouse/monocl	WB/1:5000	Abcam ab4990/AB_867494

### Image Acquisition

Images were captured using confocal laser-scanning microscope (LSM 510, Carl Zeiss GmbH, Jena, Germany), with Ar Multi-line (457, 478, 488, and 514 nm), HeNe (543 and 643 nm). Objective Plan-Neofluar ×40/1.3 Oil (Carl Zeiss GmbH, Germany) was used to capture high resolution images (pixel size = 19.76 µm^2^). The same settings were applied for all images.

### Cell density and proliferation analysis

Cell density was calculated per cell type (microglia or astrocyte) as the number of cells (OX42 or GFAP positive, respectively) divided by gap or border area. Portion of proliferating cells was calculated per cell type as the number of proliferating nuclei (Ki67 positive) of given cell type divided by the total number of that cell type (OX42 or GFAP positive) in the gap or border area. Gap areas in SW model in cell cultures were easily distinguished from the rest of the confluent parts of the coverslip. At 40x magnification the whole or majority of the gap fitted in the frame, especially at 24 and even more at 48 h, as gaps gradually closed. Gap areas were imaged and processed separately from the border images. Gap area was regarded as the space between the leading edges of astrocytes. Border area was regarded as region of cells directly encompassing the gap area. Twelve frames of gap area as well as twelve frames of border area were imaged for every coverslip (treatment group: 24 h SW, 48 h SW, 24 h SW + FnD, 48 h SW + FnD, 48 h SW: 24 h + FnD) in every genotype (TnC^+/+^, TnC^−/−^) in pre-defined positions.

### Microglial morphometric analysis

For determining morphology of microglial cells in the gap area, micrographs of cells labelled with OX-42/CD11b, GFAP and Hoechst were used. Four randomly chosen cells per culture (12 in total) were processed from gap area per treatment group (24 h SW, 48 h SW, 24 h SW + FnD, 48 h SW + FnD, 48 h SW: 24 h + FnD), per genotype (TnC^+/+^, TnC^−/−^). Images were captured using confocal laser-scanning microscope as described in Image Acquisition section.

RGB images were first transformed to 8-bit grayscale and then binarized to obtain a black and white image with established threshold (Supplementary Figures S1A–C). Images were manually edited to obtain continuous set of pixels for chosen cell (Supplementary Figure S1C). This step was done in a skilled manner by comparison to the original image in order to separate the cell processes from the surrounding cells. Cell was then cropped (Supplementary Figure S1D) and saved for further analysis as filled and outlined shape (Supplementary Figures S1E,F) (Fernández-Arjona et al., 2017). In order to analyse morphological changes of microglia in the gap area upon SW ± FnD, eleven standard parameters (Fernández-Arjona et al., 2017) were measured using ImageJ (NIH, United States of America) and FracLac plugin for ImageJ (Karperien, 2013).

Parameters were measured as follows:

1. Cell area–area of selection (µm^2^).
2. Cell perimeter—the length of the selection outline (µm).
3. Convex hull area (CHA)—area of the smallest convex polygon shape encompassing the whole selection (µm^2^).
4. Convex hull perimeter (CHP)- the length of the outline of the convex hull (µm).
5. Roughness—ratio of cell perimeter to convex hull perimeter.
6. Fractal dimension (FD)– measure of cell complexity calculated using box counting method.
7. Lacunarity—characterizes the distribution of gaps in the selection, calculated using box counting method.
8. Aspect ratio (AR)—ratio of major axis/minor axis of selection.
9. Cell circularity—(4π × cell area)/(cell perimeter)^2^. The circularity value of a circle is 1.
10. Solidity, also called density—area of the cell divided by its convex hull area.
11. Roundness—4 × area/(π × major axis^2^), or the inverse of the aspect ratio.

### Cluster analysis

Hierarchical Cluster Analysis (HCA) in SPSS was ran just to estimate the appropriate number of clusters for data sets including multiple parameters. Data sets were first standardized using Z-scores to obtain values on similar scale and Ward`s method has been used as clustering method. The number of stable clusters was used as an input for K-means cluster analysis that determined the statistical significance of parameter for clustering, assigned the cluster membership and provided distances from cluster centre for each input.

Regarding the microglia and astrocyte proliferation and density, images were sorted into three clusters based on values for all four parameters per image. For microglial morphology cells were sorted into two clusters based on all eleven parameters for each cell. Cluster members with the smallest distance from the cluster centre i.e. the most representative images were shown.

### Western blot analysis

The cultures were scraped in 150 μl of RIPA buffer, centrifuged for 10 min at 10,000 × *g* and 4°C. Supernatants were collected and the protein concentration was determined using the BCA protein assay kit, according to the manufacturer’s instruction (Thermo Fisher Scientific, United States).

The samples (10 μg of proteins) were mixed with the 6× Laemmli sample buffer (375 mM Tris-HCl, pH 6.8, 12% SDS, 60% w/v glycerol, and 0.03% bromophenol blue) containing 5% of β-mercaptoethanol. Denatured proteins were resolved on 10% SDS-PAGE gels and electro-transferred to a PVDF support membrane (Immobilon-P transfer membrane, Millipore, Merck, Germany). Membranes were blocked with 5% BSA in Tris buffer saline/Tween 20 (TBST) for 1 h, at RT. Membranes were then incubated with primary antibody overnight at +4°C, and appropriate secondary HRP-conjugated antibodies for 2 h, at RT (Table 1). Each membrane was subjected to the incubation with β-actin antibody directly conjugated with HRP for 1 h, at RT. The bands were visualized with the Clarity ECL Substrate (BioRad Laboratories, Hercules, CA, United States) and the Chemi Doc-It imaging system (UVP, Upland, CA, United States). Quantification of the bands was performed by using the ImageJ software.

### mRNA isolation and real-time PCR analysis

TRIzol reagent (Thermo Fisher Scientific, United States—Invitrogen) was used to collect the sample lysates. Further, the total RNA was extracted using phenol/chloroform procedure with ethanol precipitation. RNA concentrations were determined by measuring the absorbance at 260 nm and the purity was estimated from 260/280 nm and 260/230 nm ratios. Total RNA yield range was 0.6–1.0 ug/ml for all three cultures. Volume equivalent to 1 µg of RNA was used for reverse transcription to generate cDNA (High-Capacity cDNA Reverse Transcription Kit, Applied Biosystems, CA, US). The real-time PCR reaction mixture contained 2 μl cDNA (final concentration 20 ng/μl in 10 μl RT-PCR reaction volume), 5 μl QTM SYBR Green PCR Master Mix (Applied Biosystems, CA, US), 0.5 μl primers (100 pmol/μl, primer sequences are listed in Table 2.) and 2 μl RNase-free water. Amplification was carried out with QuantStudioTM three Real-Time PCR System (Applied Biosystems, CA, United State) under the following conditions: 10 min of enzyme activation at 95°C, 40 cycles of 15 s denaturation at 95°C, 30 s annealing at 64°C, 30 s amplification at 72°C, and 5 s fluorescence measurements at 72°C. Gene expression relative to GAPDH is presented as a log_2_-fold change of mRNA expression. To determine specificity of the PCR reaction product we performed melting curve analysis. All reactions were performed in duplicates.

**TABLE 2:** List of primer pairs for real-time PCR.

Target gene	Forward	Reverse
*Tnfα*	GCC​CAC​GTC​GTA​GCA​AAC​CAC	GGC​TGG​CAC​CAC​TAG​TTG​GTT​GT
*Il-1β*	AAA​AGC​CTC​GTG​CTG​TCG​GAC​C	TTG​AGG​CCC​AAG​GCC​ACA​GGT
*Nos2*	GGT​GTT​CTT​TGC​TTC​CAT​GCT​AAT	GTC​CCT​GGC​TAG​TGC​TTC​AGA
Arg*1*	TAA​CCT​TGG​CTT​GCT​TCG​G	GTG​GCG​CAT​TCA​CAG​TCA​C
*Gapdh*	GTT​GTC​TCC​TGC​GAC​TTC​A	TGG​TCC​AGG​GTT​TCT​TAC​TC

### Enzyme-linked immunosorbent assay

ELISA assay was performed 24 and 48 h after the SW and treatment with the addition of no SW groups. The ELISA assay was performed in a 96-well polystyrene plate coated with 100 μl of the capture antibody against IL-1β/TNF-α (88-7013-88, 1:250; 14-7423-81, 1:500, resp. ThermoFisher Scientific, United States) overnight, at RT. The wells were then washed (0.05% Tween in PBS), and incubated with 300 μl of blocking solution (5% BSA in PBS) for 1 h, at RT. After the removal of blocking solutions, 100 μl of each standard dilution point of the recombinant IL-1β/TNF-α (0.015–1 ng/ml, 39-8012-60; 0.062–4 ng/ml, 14–8321, resp, ThermoFisher Scientific, United States) were added alongside 100 μl of samples, and incubated for 2 h, at RT. Since samples were mixed cell culture media, the standard points were diluted in DMEM. After the incubation the wells were washed, and 100 μL of the detection biotinized antibody against IL-1β/TNF-α (88-7013-88, 1:250; 13-7341-81, 1:500, resp. ThermoFisher Scientific, United States) was incubated for 2 h, at RT, and washed again. For detection of biotinized antibody 100 μl of avidin-HRP (1:750, eBioscience™ 18-4100-51) was used in each well, for 20 min at RT, followed with thorough washing step. As the substrate 100 μl of 3,3′,5,5′-tetramethylbenzidine (TMB, eBioscience™ 00-4201-56) was used for 20 min, at RT. The reaction was stopped with 100 μl of 2N H_2_SO_4_. The reaction product was determined spectrophotometrically at 450 nm wavelength, while the correction absorbance was read at 570 nm.

### Data analysis and statistics

SPSS 20 software package (SPSS Inc, Chicago, IL) was used for performing two-way ANOVA and Independent-samples *t*-test (for initial density of microglia and astrocytes in cell culture). Statistically significant interactions, simple main effects, and pairwise comparisons are given in Results section text and in respective tables as *p*-values, Bonferroni-adjusted within each simple main effect. Data from each experiment is graphically presented as a box and whisker plot, where pairwise comparisons are denoted with letters in such a way that sharing the same letter implies no significant difference between those groups or genotypes within the same group.

## Results

Astrocytes and microglia are key players in reactive gliosis. In previous study, we performed scratch wound assay and tested the impact of various TnC fragments in cortical astrocyte cell cultures where this single cell type formed a monolayer. Gap closure was delayed upon addition of several TnC fragments, mainly due to decreased astrocyte proliferation and the effect of FnD was the most potent (Bijelić et al., 2021). As microglia is one of the key players in the inflammation occurring upon injury and also influences astrocyte reactivation and phenotype (Liddelow et al., 2017; Bellver-Landete et al., 2019), here we analyzed the effects of FnD upon SW in a model system of mixed cortical cultures of astrocytes and microglia. Once cultures were confluent, microglia could be found in abundance under the monolayer of astrocytes. In our experiments, the initial density of both microglia and astrocytes was higher in TnC^+/+^ than TnC^−/−^ cultures (*p = 0.001, p = 0.003*, resp. Supplementary Figure S2).

**FIGURE 1:**
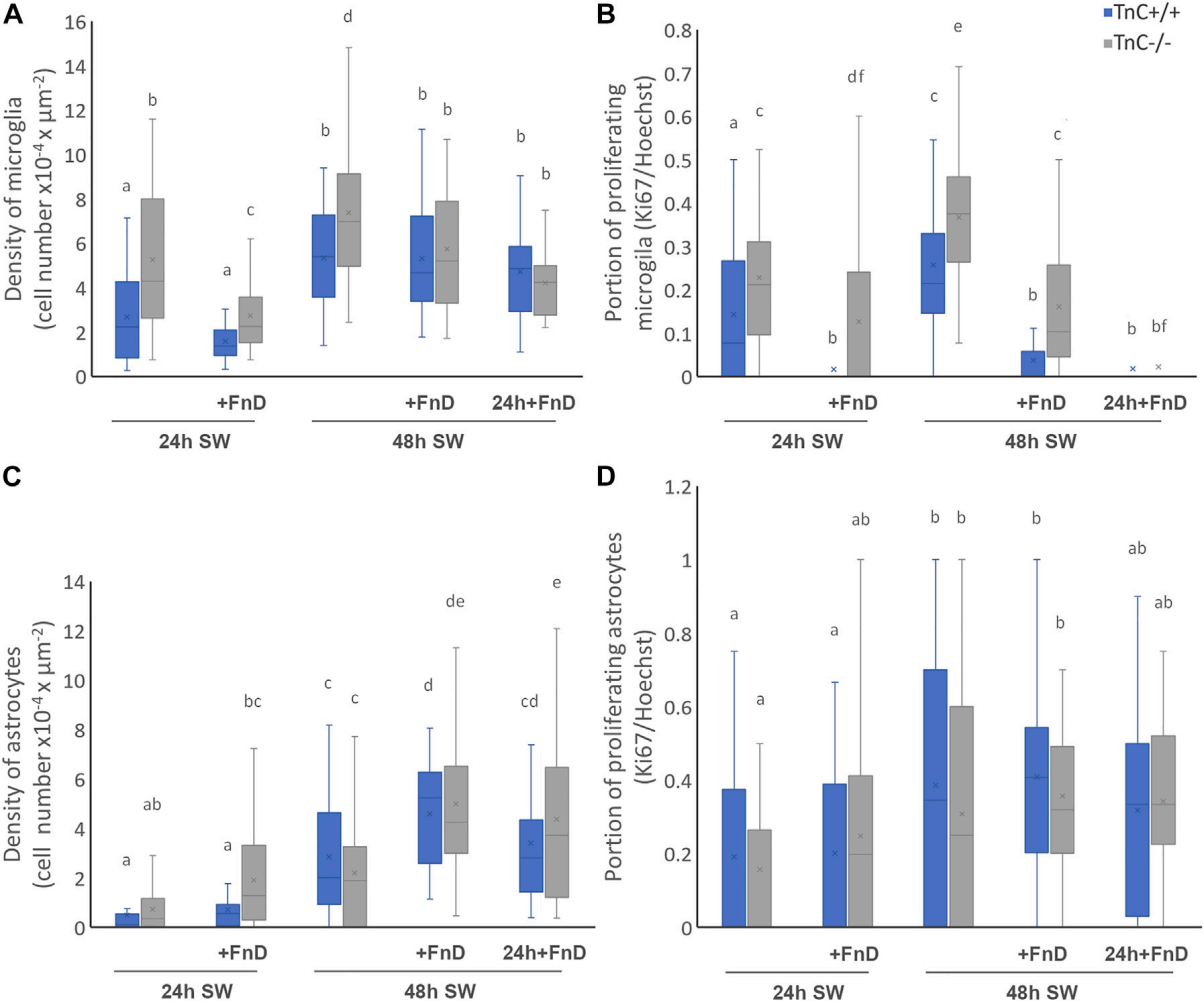
FnD reduces microglial density and proliferation, whereas it increases density of astrocytes and does not affect their proliferation in the gap area. Confocal images of cells labelled with immunofluorescent markers for microglia, astrocytes, cell nuclei and proliferation, were used for calculations of density and proliferation rates. Box—whisker plots show densities and proliferation rates of microglia [**(A,B)**, resp.) and astrocytes [**(C,D)**, resp.) in the gap area. Two-way ANOVA was used to test statistical significance. Pairwise comparisons are denoted with letters, shared letters imply no significant difference between groups and actual *p* values are indicated in the text (No. Images = 36, from three independent cultures, per treatment, per genotype).

**FIGURE 2:**
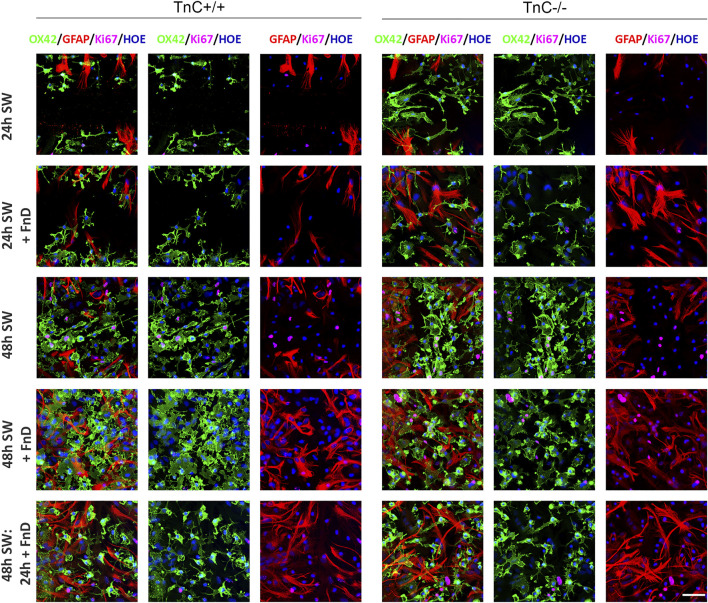
Representative micrographs of gap area depicting different densities and proliferation rates of microglia and astrocytes per treatment and genotype. Confocal images of immunofluorescently labelled microglia (OX42, green), astrocytes (GFAP, red) proliferating nuclei (Ki67, magenta) and nuclei (Hoechst, blue) within the gap area of SW per treatment/genotype. Sale bar: 50 µm.

Initially, we performed pilot experiments with SW assay (data not shown) to understand the dynamics of subsequent events in mixed cultures and decided upon focusing on two time points (24 and 48 h). In the first 24 h only a very small number of cells could be observed in the gap area, and after 48 h the cell density in the gap was so high that individual cells could not be easily distinguished. Microglial cells were the first to enter the gap area, and astrocytes were initially confounded to the border region in control groups, 24 h SW and 48 h SW. Up to 48h, the density of both cell types increased in the gap area, however, significantly more microglial cells were located in the gap area (Figures 1A,C and Figure 2). These results were in accordance with literature data, and recapitulated the order of events in the nervous tissue after injury (Beck et al., 2010; Zhou et al., 2020).

In order to examine the effects of FnD treatment on microglia and astrocytes in mixed culture upon SW we decided to assess localization, densities and proliferation rates of the two cell types. Coverslips with mixed cell cultures were scratched in cross-like manner, and at 24 and 48 h, cells were fixed and stained with OX42 and GFAP to distinguish microglia and astrocytes respectively. Hoechst was used as nuclear marker and Ki67 as marker of proliferating cells. Region between the two leading edges of astrocytes was considered as gap area. Density and proliferation were analysed for microglia and astrocytes in both gap and border areas. Changes observed in the border area are given as Supplementary Figure S3.

### FnD reduces density of microglial cells in the absence of TnC and inhibits microglial proliferation in both genotypes in the gap area

Density of microglia (Figure 1A and Figure 2) was higher in TnC^−/−^ compared to TnC^+/+^ cultures in both control groups 24 h SW and 48 h SW (*p < 0.001*), and remained higher even with the addition of FnD within the first 24 h upon SW (*p = 0.037*). In TnC^−/−^ the addition of FnD lowered density in the first 24 h (*p < 0.001*) compared to its 24 h SW control. The density of microglia was higher in 48 h SW TnC^−/−^ group compared to 24 h SW (*p = 0.001*), and FnD treated groups 48 h SW + FnD, and 48 h SW: 24 h + FnD (*p = 0.030, p < 0.001*, resp.). In group 48 h SW + FnD density was higher than in 24 h SW + FnD (*p < 0.001*), whereas the delayed addition of FnD (48 h SW: 24 h + FnD), contained densities equal to both other FnD groups. In TnC^+/+^ density of microglia was the same within 24 and 48 h groups and all of 48 h groups had higher density than at 24 h (*p < 0.001*). Both the effects of treatment and genotype were significant (*p < 0.001*), as well as their interaction (*p = 0.002*). Changes in the border zone were less pronounced than in the gap area. In TnC^−/−^ 24 h SW + FnD lowered density compared to other groups, except for 24 h delayed FnD as in gap area, and in TnC^+/+^ there were no changes in microglial density (Supplementary Figure S3A).

Percentage of proliferating microglia (Figure 1B and Figure 2) was higher in all TnC^−/−^ groups compared to TnC^+/+^ groups (*p = 0.032* 24h SW, *p = 0.002* 24 h SW + FnD, *p < 0.001* 48 h SW and 48 h SW + FnD), except for 48 h SW: 24 h + FnD. In TnC^−/−^, 24 h SW + FnD had lower portion of proliferating microglia than 24 h SW control group (*p = 0.017*)*.* 48 h SW group had statistically higher portion of proliferating microglia compared to 24 h SW and all 48 h FnD treated groups (*p < 0.001*). Delayed addition of FnD led to even more decreased proliferation rates than in 48 h SW + FnD (*p = 0.001*) but equal to 24 h SW + FnD. In TnC^+/+^ the addition of FnD also strongly diminished the portion of proliferating microglia compared to 24 h SW group (*p < 0.001*) and again 48 h SW group had statistically higher portion of proliferating microglia compared to 24 h SW (*p = 0.021*) and all 48 h FnD treated groups (*p < 0.001*). Both the effects of treatment and genotype were statistically significant (*p < 0.001*). Regarding the microglial proliferation in border region, in TnC^−/−^ only the addition of delayed FnD lowered the proliferation at 48h, and in TnC^+/+^ no impact was observed (Supplementary Figure S3B).

### FnD does not affect proliferation of astrocytes and elevates their density 48 h upon SW in the gap area

The absence of TnC had no overall impact on astrocyte density (Figure 1C and Figure 2) in control groups (24 h SW and 48 h SW). In TnC^−/−^ cultures within the first 24 h addition of FnD did not affect astrocyte density. 48 h SW group had higher densities than 24 h SW (*p = 0.024*), and even higher values were reached in 48 h SW + FnD and 48 h SW: 24 h + FnD (*p ≤ 0.001* for all comparisons). In TnC^+/+^ similarly, control and FnD-treated group had equal densities. Astrocyte density was higher, as expected, in 48 h SW group than in 24 h SW (*p < 0.001*). It was elevated even more in 48 h SW + FnD compared to 24 h SW + FnD, and 48 h SW (*p < 0.001, p = 0.003*), but it was similar to 48 h SW: 24 h + FnD. Additionally, FnD had stronger impact on density increase in TnC^−/−^ compared to TnC^+/+^ cultures both in 24 h SW + FnD (*p = 0.007*) and 48 h SW: 24 h + FnD group (*p = 0.032*). Both the effects of treatment and genotype were statistically significant (*p < 0.001, p = 0.017, resp.*) as well as their interaction (*p = 0.037*) regarding the density of astrocytes in the gap area. In border area no changes in astrocyte density were observed, regardless of time or treatment (Supplementary Figure S3C).

The absence of TnC also had no impact on overall proliferation rate of astrocytes (Figure 1D and Figure 2) nor did the addition of FnD. In TnC^−/−^ only 48 h SW had higher proliferation rates than 24 h SW (*p = 0.041*). In TnC^+/+^ cultures, portion of proliferating astrocytes was equal within 24 and 48 h groups. 48 h SW had higher proliferation values than 24 h SW (*p = 0.017*), and 48 h SW + FnD than 24 h SW + FnD (*p = 0.019*). Only the effect of treatment was significant (*p < 0.001*). When it comes to the border region, FnD lowers proliferation rates in both genotypes at 48 h, except for 48 h SW: 24 h + FnD in TnC^−/−^ (Supplementary Figure S3D).

### Gap areas were smaller within the first 24 h in the absence of TnC, while FnD had no effect

In wild-type cultures gap areas (Supplementary Figure S4) were smaller at 48 h than at 24 h as expected. The absence of TnC stimulated faster gap closure within the first 24 h than in TnC^+/+^, whereas afterwards, all gap areas were similar in both genotypes. The addition of FnD did not impact significantly gap areas.

### Responses of mixed cultures to SW ± FnD were distributed into three clusters based on cell density and proliferation

**FIGURE 3:**
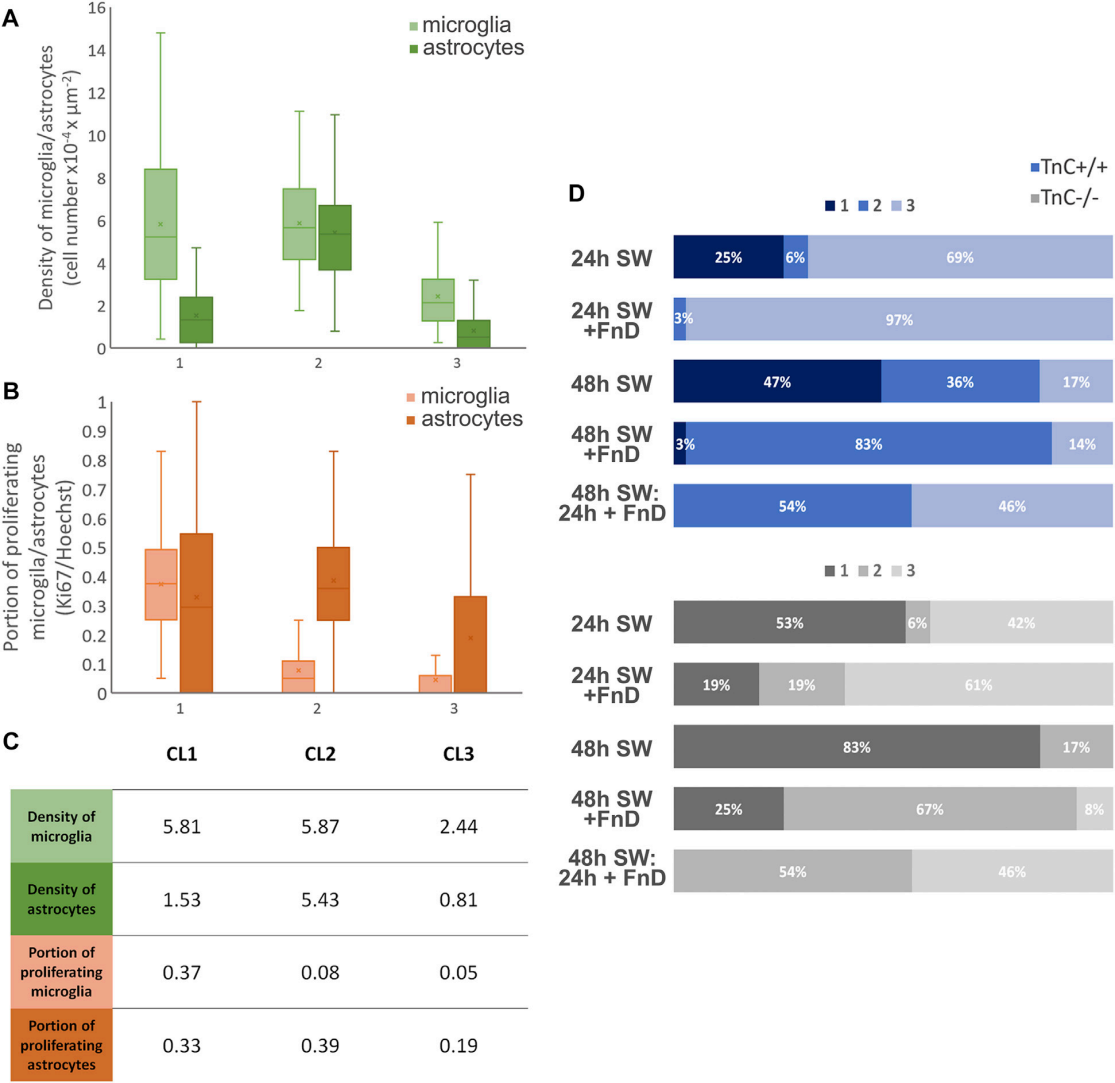
Timing and duration of FnD treatment induces different values of microglia and astrocyte proliferation and density in the gap area per genotype. Responses of microglia and astrocytes in mixed cultures to SW ± FnD were distributed into three clusters based on cell density and proliferation. Range of values for densities of microglia and astrocytes per cluster are given as box—whisker plots **(A)**. Range of values for proliferation rates of microglia and astrocytes per cluster are given as box-whisker plots **(B)**. Average values per parameters which represent cluster centres are given in table **(C)**. 100% stacked bar chart showing percentages of cluster membership per each group in respective genotype. HCA and K-means cluster analysis were used. Clusters were marked as 1, 2 and 3 **(A,B,D)** and CL1, CL2 and CL3 **(C)** (No. Images = 36, from three independent cultures, per treatment, per genotype).

Four parameters, density and proliferation of astrocytes and microglia were first analysed separately as shown above. Next, in order to see the overall effect of SW ± FnD, values for all four parameters per image were observed together. Using HCA and K-means cluster analysis based on these four parameters images were sorted into three clusters (*p* < 0.001; Figure 3).

Values for densities of microglia and astrocytes per cluster are given in Figure 3A, and values for proliferation rates of two cell types per cluster are in Figure 3B. Average values for each parameter denoting the respective cluster center are given in Figure 3C. The first cluster was characterised by higher densities of microglia with high proliferation rates, and medium densities of astrocytes with higher proliferation rates. The second cluster also had higher densities of microglia, but with lower proliferation rates, much higher densities of astrocytes than in the other two clusters and high astrocyte proliferation rates. In the third cluster, densities for both cell types as well as their proliferation rates were the lowest compared to the other two clusters. In Figure 3D distribution of images for each treatment within the three clusters is given per respective genotype.

In the first 24 h, the absence of TnC led to equal distribution of images in clusters one and three compared to the wild-type cultures where cluster three was the most represented (Figure 3D). Without TnC, densities and proliferation rates of microglial cells were more frequently higher compared to the wild-type cultures. The difference was not so obvious with astrocytes, as they were sparse in the gap area within the first 24 h (Figure 2). The addition of FnD shifted cluster membership to the cluster three, implicating lowering of microglial density and proliferation rates. The effect was more pronounced in wild-type cultures, implying synergistic effect with the endogenously expressed TnC. At 48 h absence of TnC strongly increased frequency of the first cluster with the highest levels of microglial proliferation and densities, whereas in the TnC^+/+^ cultures clusters one and two were equally represented. The addition of FnD boosted the second cluster membership due to higher densities of astrocytes in the gap area at 48h, as it can be seen in Figure 2. In case of the delayed addition of FnD (48 h SW: 24 h + FnD) images were equally distributed in the second and third cluster, with no images in the first cluster. The observed effect was balancing the effects of 24 and 48 h administered FnD.

### FnD induced activated microglial morphology in the gap area

**FIGURE 4:**
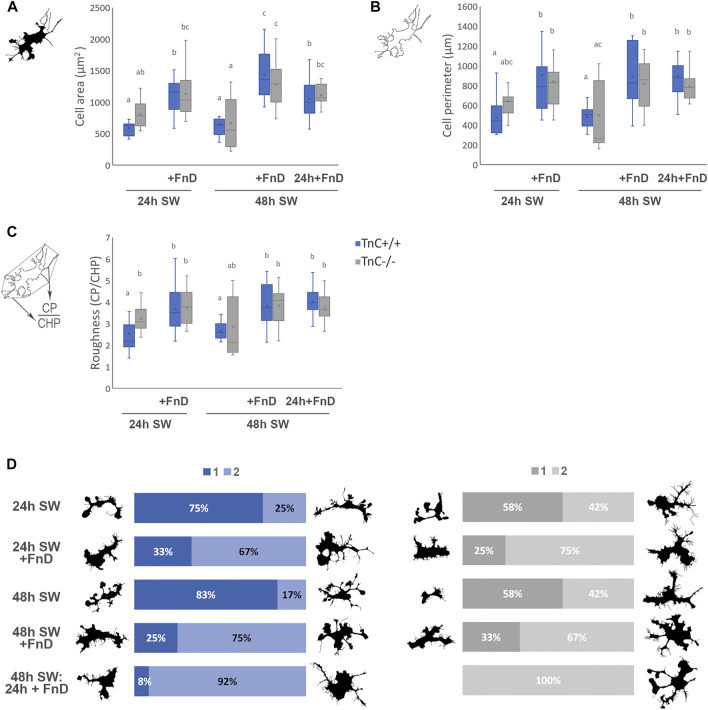
FnD treatment induces activated microglial morphology in the gap area. Box—whisker plots of microglial cell area **(A)** cell perimeter **(B)** and cell roughness **(C)** are shown. Two-way ANOVA was used to test statistical significance. Pairwise comparisons are denoted with letters, shared letters imply no significant difference between groups. Actual *p* values are indicated in the text. Microglial cells were sorted into two clusters based on eleven measured morphological parameters per cell (main text Table 3). 100% stacked bar chart representing percentages of cluster membership per each group in respective genotype is shown including most representative cell shapes-cluster members with the smallest distance from the cluster centre **(D)**. Clusters were marked as 1 and 2 **(D)** (No. Microglial cells = 12, from three independent cultures, per treatment, per genotype).

Eleven morphological parameters were analyzed for each chosen cell in the gap area as described in the Material and Methods section. Cells were clustered based on all eleven parameters in two clusters (*p < 0.001* for all except for Solidity, *p = 0.020*; Figure 4D; Table 3). For most of the parameters average values were smaller in the first cluster, except for cell circularity, aspect ratio and solidity, and filled shapes of the representative cells per each cluster are displayed in Figure 4D. The absence of TnC increased membership to the second cluster compared to the wild-type cultures, but cluster one was still predominant. With the addition of FnD cluster two became predominant. Upon FnD treatment thin elongated cell processes connected and the space in between was filled with cytoplasm thus creating swollen like cell protrusions and larger cell areas in total. Also, cells were covered in thin filopodia much more which increased significantly cell perimeters and also influenced higher roughness index. Three parameters, with the most pronounced statistically significant differences, cell area, perimeter and roughness were presented in Figures 4A–C, while the other eight are given as Supplementary Figure S5.

**TABLE 3:** Cluster centres for microglial shape descriptors.

	Cell area	Cell perim.	Circ.	AR	Roundn.	Solidity	Roughn.	FD	Lac.	CHA	CHP
CL1	708.46	453.57	0.05	2.44	0.49	0.40	2.56	1.21	0.29	1,803.64	174.84
CL2	1,174.91	903.94	0.02	1.68	0.64	0.35	3.98	1.30	0.33	3,421.44	225.67

### Addition of FnD increased cell area, and the absence of TnC had the same tendency

Microglial cell areas (Figure 4A) were on average similar in both genotypes, however in the absence of TnC there was a tendency towards larger areas. Hence the addition of FnD within the first 24 h made no significant impact in TnC^−/−^ but it increased cell areas in TnC^+/+^ compared to the control group (*p = 0.001*). At 48 h in control groups cell areas were similar to those in 24 h SW group in both genotypes. In TnC^−/−^ genotype both 48 h SW + FnD and 48 h SW: 24 h + FnD induced bigger cell areas than 48 h SW control group (*p < 0.001*, *p = 0.003*, resp.), and all FnD-treated groups were equal to each other. In TnC^+/+^ 48 h SW + FnD treatment induced bigger cell areas compared to 48 h SW and the other two FnD-treated groups 24 h SW + FnD and 48 h SW: 24 h + FnD (*p < 0.001, p = 0.045*, *p = 0.017*, resp.). 48 h SW: 24 h + FnD had also bigger cell areas than 48 h SW (*p* = 0.003), and on average the same as 24 h SW + FnD. On overall only treatment was statistically significant (*p < 0.001*).

### Addition of FnD increased microglia cell perimeters

Microglial perimeters (Figure 4B) were similar between control groups of both genotypes, although in the absence of TnC tendency to larger cell perimeters has been observed. In TnC^−/−^ cultures the addition of FnD did not significantly alter cell perimeter within the first 24h, but increased it compared to 48 h SW control in both 48 h SW + FnD and 48 h SW: 24 h + FnD groups (*p = 0.010*, *p = 0.023*, resp.). In all FnD-treated groups in TnC^−/−^ genotype, cell perimeters were the same on average. As was the case with cell area, FnD promoted larger cell perimeters in TnC^+/+^ at 24 h compared to the 24 h SW (*p = 0.023*), and at 48 h both 48 h SW + FnD and 48 h SW: 24 h + FnD had bigger perimeters than 48 h SW (*p < 0.001*). Here also there were no differences amongst FnD-treated groups.

### Addition of FnD increased microglial cell roughness in wild-type cultures, whereas TnC^−/−^ cells already exhibited increased cell roughness

Lack of TnC stimulated higher roughness values of microglial cells (Figure 4C) at 24 h in control 24 h SW group compared to TnC^+/+^ cultures (*p = 0.008*). FnD had no additional impact in TnC^−/−^ cultures. On the other hand, in TnC^+/+^ 24 h SW + FnD had higher roughness values than 24 h SW (*p = 0.001*). 24 h SW and 48 h SW did not differ, but 48 h SW + FnD and 48 h SW: 24 h + FnD had higher roughness values than 48 h SW (*p = 0.009*, *p = 0.001*).

### FnD upregulated the levels of pro-inflammatory cytokines TNF-α and IL-1β

**FIGURE 5:**
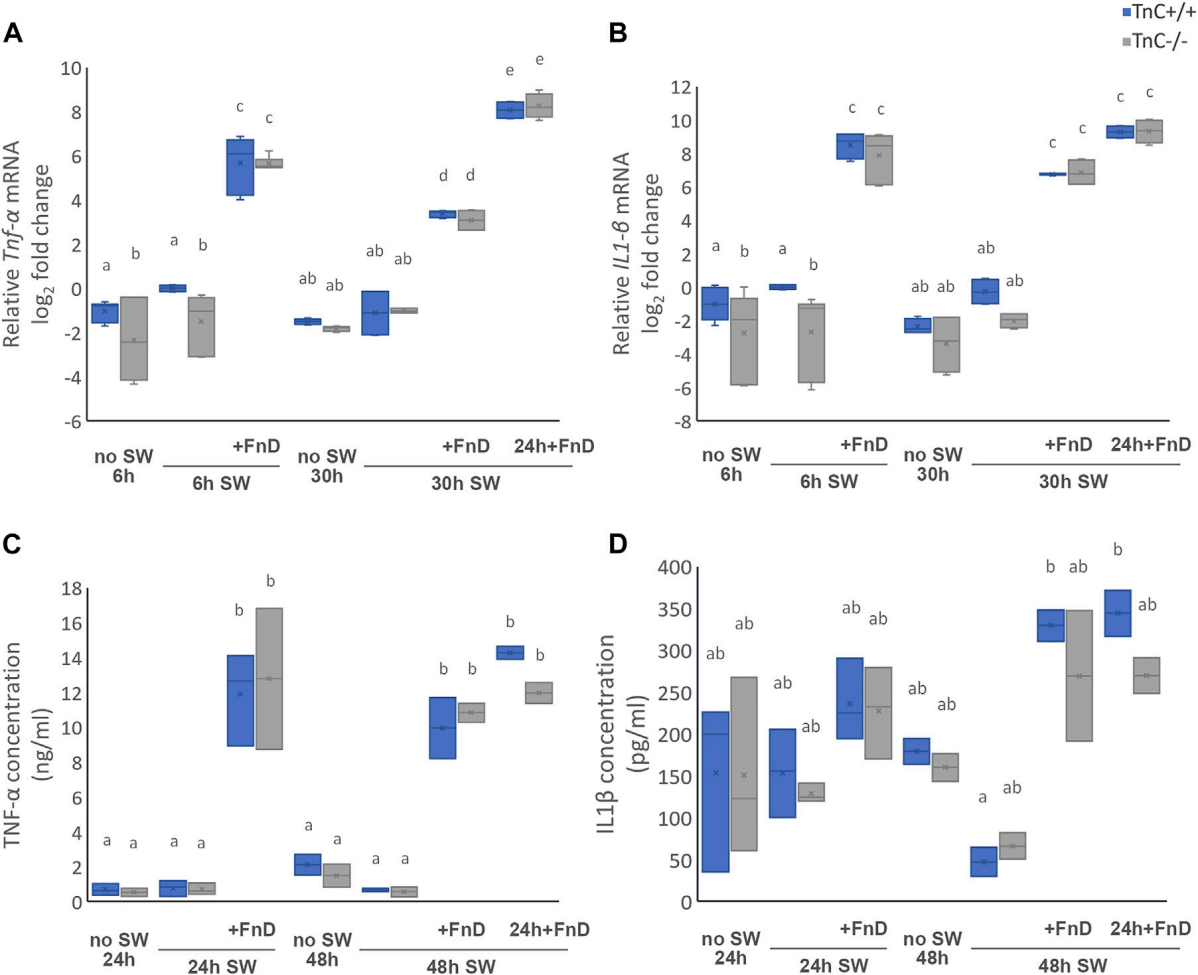
FnD treatment upregulates levels of pro-inflammatory cytokines TNF-α and IL-1β. Relative *Tnfa* mRNA log2 fold change **(A)** and relative *IL1b* mRNA log2 fold change **(B)** are shown as box-whisker plots. Concentrations of cytokines released into the medium are shown as box-whisker plots for TNF-α **(C)** and IL-1β **(D)**. Two-way ANOVA was used to test statistical significance. Pairwise comparisons are denoted with letters, shared letters imply no significant difference between groups. Actual *p* values are indicated in the text (n = 3 independent cultures, performed in duplicate).

In order to further investigate the reactivation profile of glial cells upon SW and FnD treatment, Tnf-*α* and IL-1β were examined at the level of mRNA expression and concentrations of these cytokines released into the medium (Figure 5). In the absence of TnC levels of *Tnfα* mRNA were lower in the intact 6 h no SW and 6 h SW control groups than in TnC^+/+^ genotype (*p = 0.008, p = 0.003*, resp.; Figure 5A). No difference could be observed later between the groups in the two genotypes. Within genotypes, no SW and SW groups were on average similar at 6 and 30h, and all of the FnD-treated groups had significant increase of mRNA levels compared to all untreated groups (*p < 0.001*). In both genotypes the greatest increase was reached in group 30 h SW: 24 h + FnD compared to 6 h SW + FnD and 30 h SW + FnD (*p ≤ 0.001*). 6 h SW + FnD had also significantly higher increase than 30 h SW + FnD in TnC^+/+^ and TnC^−/−^ (*p = 0.002, p < 0.001,* resp.). On overall, both genotype and treatment were statistically significant (*p = 0.032, p < 0.001,* resp.) regarding the levels of *Tnfα* mRNA.

mRNA levels of *Il1b* were also higher only initially in TnC^+/+^ groups 6 h no SW and 6 h SW than in TnC^−/−^ (*p = 0.029, p = 0.001*, resp. Figure 5B). Again, no differences were observed among no SW and SW groups within genotypes at 6 and 30 h, and all FnD-treated groups were equal to each other but showed significant increase of mRNA levels of *Il1b* compared to untreated groups (*p < 0.001*). Statistical analysis of *Il1b* mRNA levels showed that both genotype and treatment were significant (*p = 0.002, p < 0.001,* resp).

Concentrations of cytokines released in medium were assessed by ELISA. Regarding the concentration of Tnf-α, no differences were observed between the genotypes, and only treatment was statistically significant (*p < 0.001,* Figure 5C). No SW and SW groups had on average the same concentrations in all time points, whereas all FnD-treated groups had higher concentrations (*p ≤ 0.001*) and were equal mutually.

Within the first 24 h in all of the groups in both genotypes, similar concentrations of IL-1β were released into the medium (Figure 5D). At 48 h the concentration of IL-1β did not change in no SW groups in both genotypes and remained equal to concentrations released at 24 h. On the contrary, discrepancies were observed in TnC^+/+^ between 48 h FnD-treated groups (48 h SW + FnD and 48 h SW: 24 h + FnD) and 48 h SW group since concentrations decreased in untreated and increased in treated groups (*p = 0.004, p = 0.002,* resp*.*). The same tendency was seen in TnC^−/−^ cultures, however not statistically significant. On overall, only treatment was significant (*p < 0.001*).

### FnD treatment strongly upregulated iNOS expression, consistent with pro-inflammatory phenotype

**FIGURE 6:**
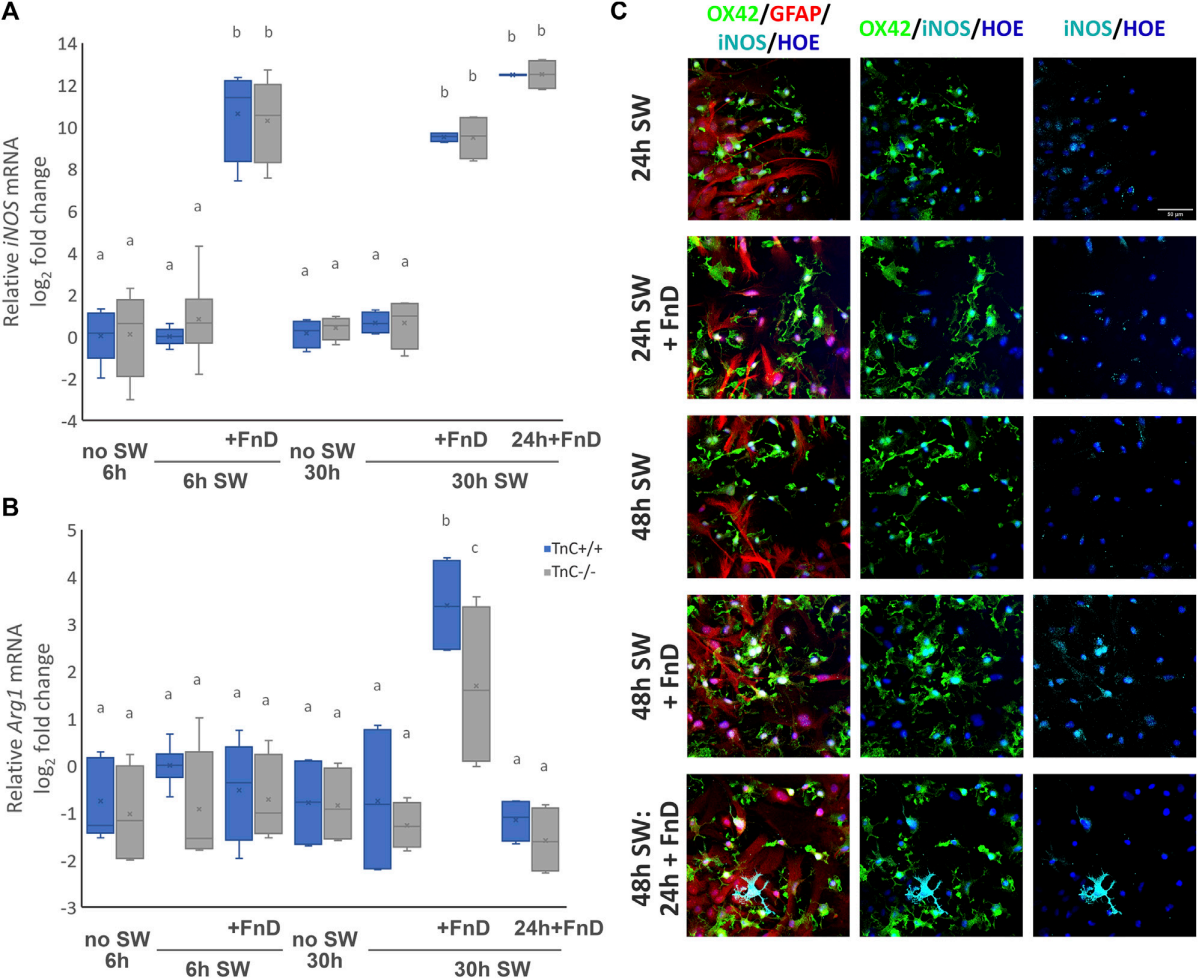
FnD treatment upregulates expression of *iNOS* at all timepoints and *Arg1* at 30h upon SW. Box—whisker plots of relative *iNOS* mRNA log_2_ fold change **(A)** and relative Arg*1* mRNA log2 fold change **(B)** are shown. Two-way ANOVA was used to test statistical significance. Pairwise comparisons are denoted with letters, shared letters imply no significant difference between groups. Actual *p* values are indicated in the text (n = 3 independent cultures, performed in duplicate). Representative micrographs of immunofluorescently labelled OX42 (green), marker of microglia, GFAP (red) marker of astrocytes, iNOS (cyan) and Hoechst (blue) nuclear marker **(C)**. Strong iNOS immunoreactivity was observed on some microglial cells in group 48 h SW: 24 h + FnD in both genotypes in the gap area (only wild-type shown).

Regarding the *iNOS* mRNA expression levels (Figure 6A), all no SW and SW groups had lower relative mRNA log_2_ fold change compared to all of FnD-treated groups (*p < 0.001*). There were no differences among genotypes. Only treatment was statistically significant (*p < 0.001*). In group 48 h SW: 24 h + FnD in both genotypes, strong iNOS immunoreactivity was observed on some microglial cells mainly in the gap area labelling cell soma and processes as shown in Figure 6C (only wild-type shown).

Relative Arg*1* mRNA log_2_ fold change (Figure 6B) was on average equal in all groups in both genotypes except for the 30 h SW + FnD group. In TnC^+/+^, this group exhibited the highest levels of Arg *1* mRNA compared to all the other groups (*p < 0.001*). This elevation was also significantly higher than in the same group in TnC^−/−^ genotype (*p = 0.019*). In TnC^−/−^, in 30 h SW + FnD group Arg*1*mRNA levels were upregulated also significantly compared to 6 h SW + FnD, 30h SW, 30 h SW: 24 h + FnD (*p = 0.010, p = 0.002, p = 0.001*). Both genotype and treatment were significant (*p = 0.022, p < 0.001,* resp).

### FnD elevated the ratio of precursor to mature isoform of β1 integrin

**FIGURE 7:**
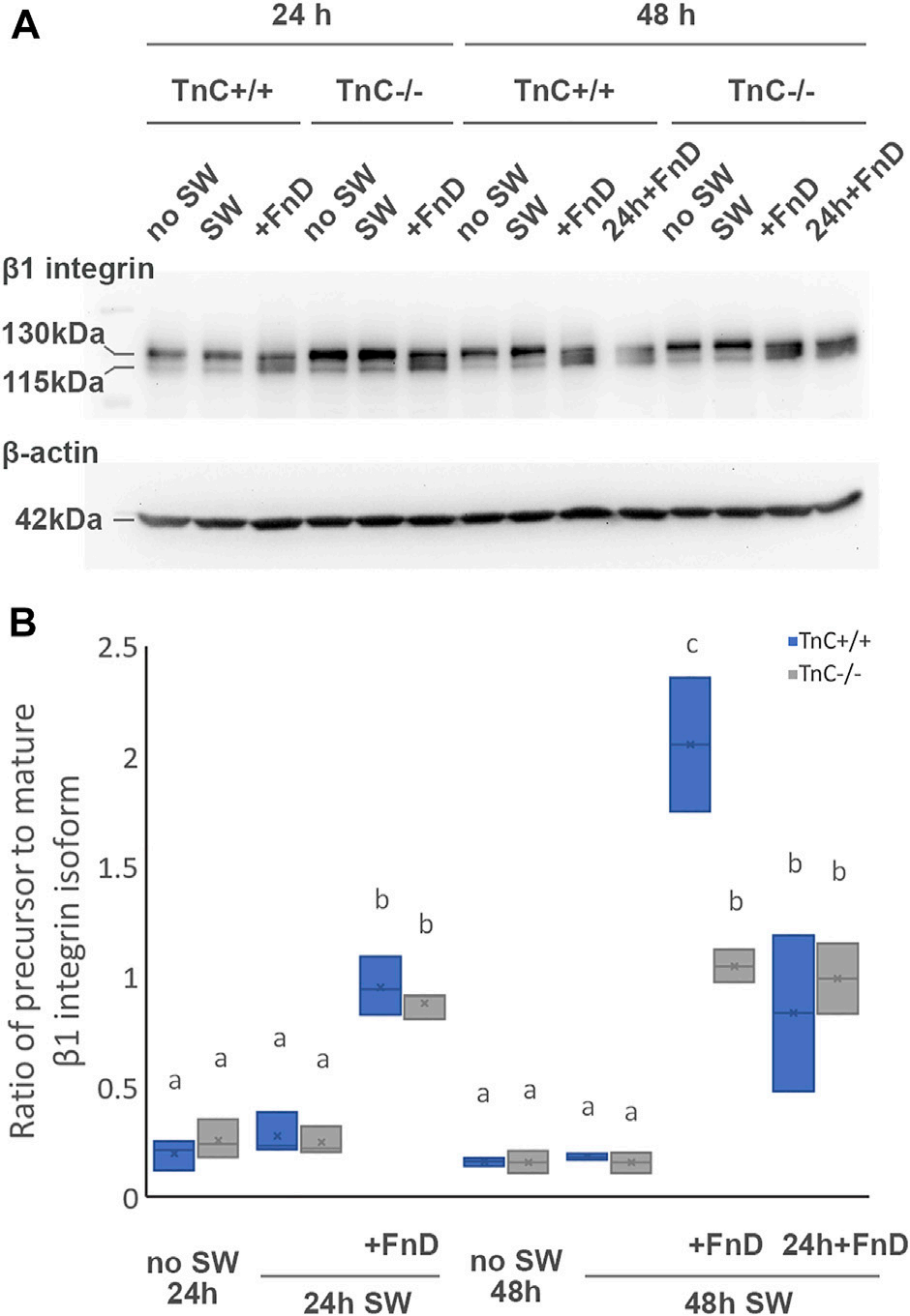
FnD treatment increases the ratio of precursor to mature β1 integrin isoform. Representative Western blot image is shown for β1 integrin and respective β-actin **(A)**. Box—whisker plots of ratio of precursor to mature β1 integrin isoform are shown **(B)**. Two-way ANOVA was used to test statistical significance. Pairwise comparisons are denoted with letters, shared letters imply no significant difference between groups. Actual *p* values are indicated in the text (*n* = 3 independent cultures).

β1 Integrin expression was analyzed using Western blot. Both the totally glycosylated mature and partially glycosylated precursor bands were observed at 130 and 115kDa, respectively (Figure 7A, Supplementary Figure S6). The optical density of the protein bands was analyzed using ImageJ software and the ratio of precursor to mature isoform was calculated (Figure 7B). All no SW and SW groups in both genotypes had similar ratios, with greater expression of mature form. However, all of the groups treated with FnD had higher expression of precursor isoform. The highest values for precursor form were reached in 48 h SW + FnD, and in particular in TnC^+/+^. In TnC^+/+^, 24 h SW + FnD had higher ratio than 24 h SW (*p = 0.002*). Both 48 h present D and delayed FnD increased ratio compared to 48 h SW (*p < 0.025, p < 0.001*, resp). 48 h SW + FnD had also higher precursor values than 24 h SW + FnD (*p < 0.001*). In TnC^−/−^ 24 h SW + FnD had higher ratio than 24 h SW (*p = 0.005*), and both 48 h present D and delayed FnD compared to 48 h SW (*p = 0.001, p = 0.002*, resp). In 48 h SW + FnD, higher values were observed in TnC^+/+^ than in TnC^−/−^ (*p < 0.001*). Both the genotype and treatment were statistically significant as well as their interaction (*p = 0.043, p < 0.001, p = 0.002,* resp.). TnC, and in particular FnD did not induce any changes regarding β3 integrin in respect to the bands position or intensity (data not shown).

### FnD causes the appearance of specific P2Y12R monomeric bands

**FIGURE 8:**
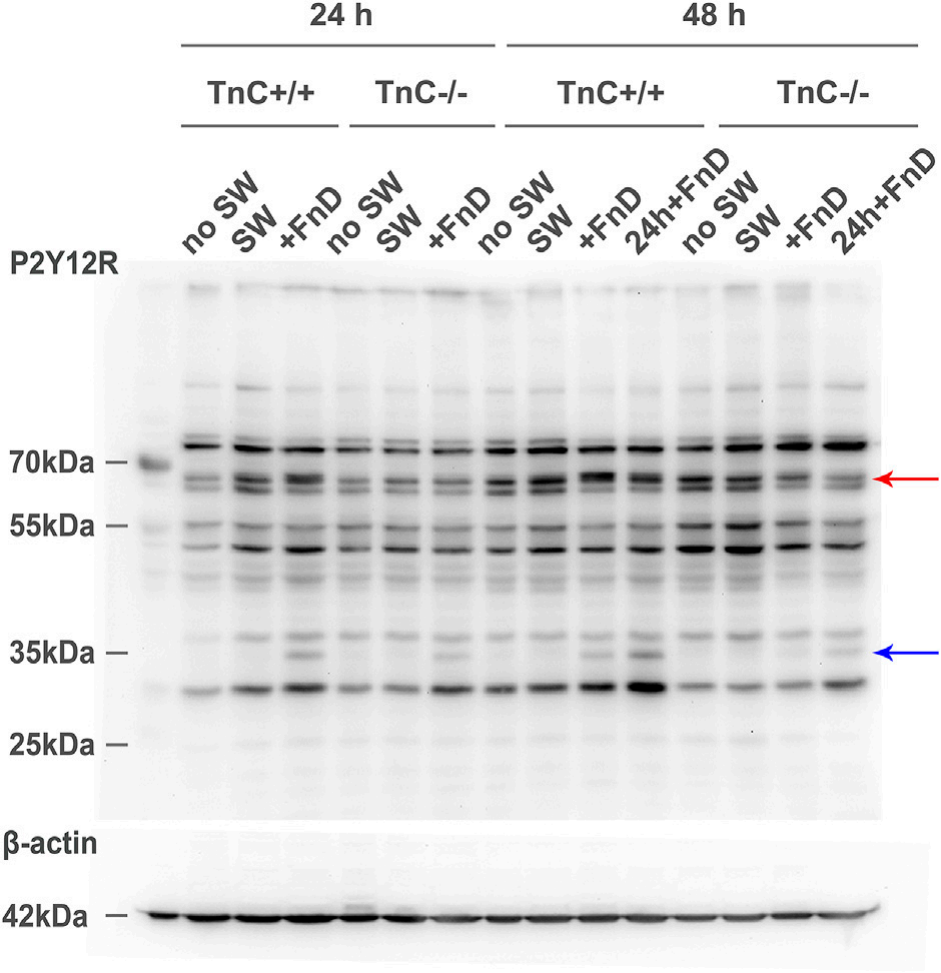
Appearance of specific P2Y12R monomeric bands upon FnD treatment. Representative Western blot image is shown for P2Y12R and respective β-actin. Blue arrow indicates positions of monomeric bands at ∼35 kDa. Additional bands appear only with FnD treatment. Red arrow indicates two bands at the level of dimers ∼60 kDa of P2Y12R. In groups treated with FnD, more intense upper band could be seen (more pronounced in TnC^+/+^) (*n* = 3 independent cultures).

The expression of protein P2Y12R was assessed with Western blotting technique. In all groups treated with FnD regardless of time point, the presence of bands at approximately 35 kDa was observed that corresponds to monomer of P2Y12R (Figure 8). Additionally, in the range of P2Y12R dimers (∼60 kDa), two bands were visible and in FnD-treated groups the upper band appeared to have stronger intensity, and the effect was more prominent in TnC^+/+^ cultures. Our results suggest that upon FnD treatment *de novo* monomeric P2Y12R bands appear whereas rearrangements in band representation occur in the range of dimer forms. We examined also expression of P2Y13R, but all bands appeared uniform regardless of presence of TnC or FnD (data not shown).

## Discussion

Our results indicate that FnD influences the involvement of microglia and astrocytes in wound healing. FnD treatment decreased density and proliferation of microglia and stimulated migration of astrocytes into the injury. Microglial cells exhibited activated morphology and upregulated levels of proinflammatory markers. Production of proinflammatory cytokines increased in FnD-treated cultures and shifts were observed in the expression levels of isoforms of receptors involved in cell morphology and migration.

Expression and secretion of TnC by cultured astrocytes was examined in several studies. Nishio et al., showed biphasic secretion of TnC into the medium in induced astrogliosis model in long-term primary cultures with the first higher peak at 4 h and the second lower at 48 h (Nishio et al., 2003), and in the following study, the release of the largest TnC isoform by cultured astrocytes upon SW reaching peak 12 h after the wounding (Nishio et al., 2005). Based on all observed differences between the two genotypes in our experiments, we can conclude that certain isoform of TnC was synthesised and secreted in TnC^+/+^ cultures up to 24 h after SW. As upregulation of TnC expression is expected after injury, we considered the delayed addition of FnD to be more physiologically relevant approximation of the *in vivo* situation. This postponed addition induced potentiation of the observed effects in the absence of TnC, while it had no significant impact on wild-type cultures.

Cell migration and proliferation are principal mechanisms through which tissue is repaired and repopulated upon injury (Cavanagh, 1970; Latov et al., 1979; Giordana et al., 1994; Rhodes et al., 2003; Midwood and Orend, 2009; Liddelow et al., 2020). TnC has been shown to have both adhesive and antiadhesive effects for neuronal and diverse non-neuronal cell types due to diverse functions of its distinct domains and existence of multiple binding partners with various downstream intracellular signalling pathways (Chiquet-Ehrismann, 1991; Scholze et al., 1996). The coexistence of both binding and repellent functions is the basis of cell migration process. Fn1-3 repeats together with Fn7,8 and FG motif at the carboxy terminal were recognized as cell attachment promoting sites for glial cells (Gotz et al., 1996), while, EGF-like repeats, Fn4-5 and the alternatively spliced regions were attributed with anti-adhesion properties (Prieto et al., 1992; Gotz et al., 1996; Scholze et al., 1996). It is already well established that alternatively spliced domains of TnC induce dose-dependent reduction of focal adhesion (Murphy-Ullrich et al., 1991), through binding to annexin II (Chung and Erickson, 1994) thus enhancing cell migration in endothelial cell culture wound assay (Chung et al., 1996).

Dual effects of TnC have also been reported regarding cell proliferation as it has been known to promote proliferation in cancer tissues, but sustain or inhibit it in fibroblasts (Yoshida et al., 2015). Whereas constitutively expressed domains Fn1-5 and 6-8 were shown to promote adhesion and proliferation of human hematopoetic cells (Seiffert et al., 1998), in our previous study we observed decrease of astrocyte proliferation in border region of wound upon treatment with exogenously added FnA, FnD, Fn6-8, and EGFL (Bijelić et al., 2021). Crossin showed that exogenously added TnC inhibited cell proliferation of fibroblasts *in vitro* even in the presence of mitogenic stimulators such as growth factors and tumor promoters, by inducing early inhibition of the intracellular alkalinization that occurs upon mitogenic stimulation (Crossin, 1991). Another study reported that TnC inhibited adhesion and proliferation of anchorage-dependant fibroblasts by reducing cyclin-dependent kinase 2 activity, and arresting cells in G1/S phase, interfering with fibronectin-syndecan-four interactions (Orend et al., 2003).

In our experiments the absence of TnC in TnC^−/−^ cultures promoted proliferation and likely migration of microglia into the wound area as their density was greater than in TnC^+/+^ cultures, and no such effects were observed in the border region. On the other hand, the lack of TnC had no significant impact on the density and proliferation rates of the astrocytes within the gap region, but in the border region density of astrocytes increased within the first 24 h, without changes in proliferation rates, thus causing faster gap closure during this period.

Regarding the FnD treatment, whereas microglial proliferation was decreased in the gap region during the entire 48 h, astrocytic proliferation was not affected. Yet, proliferation was decreased for both cell types in the border region during the second 24 h upon SW. FnD decreased microglial density in the gap throughout the 48 h in TnC^−/−^ cultures, through diminished proliferation and possibly through decreased migration, but it had no impact on the astrocyte density during the first 24 h, and strongly promoted their migration into the gap in the second 24 h upon SW.

One of the most important binding partners of TnC are integrins, transmembrane receptors involved in cell-cell and cell-ECM interactions that modulate cell growth, adhesion, migration, proliferation, signalling and cytokine production (Sriramarao et al., 1993). The integrin heterodimers of α2/7/8/9β1 and αvβ1/3/6 are known to mediate signals between cells and TnC (Yoshida et al., 2015). Interactions with α9β1 and αVβ3 were found to promote cell proliferation, and migration was found to be both stimulated and inhibited as a consequence of TnC-integrin interactions. α7β1 was shown to bind to specifically FnD and promote the outgrowth of cerebellar granule neurons (Tucker and Chiquet-Ehrismann, 2015). We observed 2 bands of β1 integrin that corresponded to β1 integrin isoforms described in literature as 115 kD partially glycosylated precursor and a 130 kD fully glycosylated mature form and the addition of FnD application induced significantly higher expression of the precursor isoform. One study reported that (She et al., 2010) overexpression of Nm23 metastatic suppressor gene on hepatocarcinoma cells resulted in these two typical β1 integrin bands, and decreased ratio of mature to precursor integrin isoforms. This was due to the impaired glycosylation of β1 integrin precursor that down-regulated β1integrin expression on cell surface, and resulted in the reduced interaction with fibronectin, impaired focal adhesion, actin cytoskeleton formation and cell migration (Isaji et al., 2009; She et al., 2010; Hang et al., 2017; Moreno-Layseca et al., 2019). Syndecan—four and annexin II which are also binding partners of TnC have been identified as some of the key controllers of the integrin recycling (Morgan et al., 2013; Rankin et al., 2013). Through activity of proteolytic enzymes and conformational changes, otherwise hidden sites of extracellular matrix (ECM) proteins can be cleaved into biologically active fragments thus called matricryptins. Fibronectin (FN) is known to have FNIII14, a 22-mer matricryptin that suppresses β1 integrin-mediated cell adhesion to FN, while similar matricryptin in TnC, TnIIIA2 stimulates cell adhesion promoting interaction of β1 integrin with syndecan—4 (Saito et al., 2007).

Another important TnC binding partner is TLR4 and through this interaction TnC regulates chemotaxis, phagocytosis, proinflammatory cytokine production (Midwood et al., 2009; Marzeda and Midwood, 2018; Haage et al., 2019). By activating TLR4, TnC promotes secretion of pro-inflammatory mediators (IL-6, IL-8, and TNF) in various cell types like macrophages, dendritic cells, fibroblasts and chondrocytes (Zuliani-Alvarez et al., 2017). Recently, it has been shown that this activation also leads to inflammasome priming and subsequent production of IL-1β and IL-18 (Broz and Dixit, 2016). We showed in our previous study upregulation of pro-inflammatory Tnf-α and IL-1β levels upon scratch wound and addition of FnA, FnD, Fn6-8, and EGFL in cultured astrocytes (Bijelić et al., 2021). In the current study, the addition of FnD strongly stimulated the upregulation of mRNA levels of Tnfα and IL-1β and the release of cytokines into the medium implying the reactive and inflammatory phenotype of microglia and astrocytes (Walker et al., 2014; Orihuela et al., 2016).

Production of inducible nitric oxide synthase (iNOS) is one of hallmarks of pro-inflammatory microglial phenotype whereas arginase 1 (Arg1) is present more in pro-resolving phenotype (Cherry et al., 2014). Activation of TLR4 has been shown to upregulate the expression of NADPH oxidase and production of *iNOS* (Deng et al., 2015). Our experiments demonstrated the increase of mRNA levels of iNOS upon addition of FnD and in case of delayed FnD addition even strong immunofluorescence present in the whole microglial cells in the gap area. *Arg1* mRNA levels were on the other hand elevated only in groups treated with FnD for 48 h and more in wild-type cultures indicating maybe that at that point also pro-resolving phenotype starts to appear.

We have further demonstrated the development of inflammatory phenotype of microglia in the gap area through morphological changes that occur upon FnD treatment. The addition of FnD promoted formation of swollen-like thick processes and greater cell coverage by thin filopodia that increased significantly cell areas and perimeters and affected cell roughness index. These thin processes were shown to be actin-dependent filopodia responsible for faster cue sensing in the discrete regions (Bernier et al., 2019). It should also be emphasized that morphometric differences induced by FnD were less significant compared to the SW controls in TnC^−/−^ cultures, especially in the first 24 h indicating that the absence of tenascin C alone promotes enlarged cells with more processes. The usual role of microglia is the surveillance of the brain parenchyma through continuous processes movement (Nimmerjahn et al., 2005). In response to the injury microglia undergoes changes in morphology ranging from enlarged cell bodies with thick processes to smaller, mainly ameboid morphology resulting from the activation of P2Y receptors by released ATP (Davis et al., 1994; Davalos et al., 2005; Morrison et al., 2017). P2Y12R was shown to be important for polarization, migration and process extension towards chemotaktins released in the injury site and is downregulated upon microglial activation (Haynes et al., 2006). Nasu-Tada et al. hypothesised that activation of P2Y12 receptors in injury by ATP and ADP causes shifting of β1 integrin to membrane ruffle regions which decreases the adhesion and proliferation and promotes the migration into the injury (Nasu-Tada et al., 2005). P2Y12 receptor has been extensively studied also in platelets as being essential for their activation. In platelets P2Y12 receptors are located mainly in lipid rafts as homooligomers in physiological state, however addition of antithrombotic drug, clopidogrel was shown to induce its dissociation into non-functional dimers and monomers that were sequestered outside the lipid rafts (Savi et al., 2006). Functional oligomers of P2Y12R have been shown to be approximately 220kDa, while monomeric and dimeric forms can be observed at approximately 40 and 80 kDa, respectively (Zhang et al., 2015). In our study, additional P2Y12R bands at 35 kDa, corresponding to monomeric forms appeared only with the FnD treatment. In the range of dimeric forms, stronger intensity of upper bands was observed also only in FnD-treated groups, and the effect was more pronounced in TnC^+/+^ cultures. Thus, weather FnD leads to *de novo* synthesis of P2Y12R, or it has a role in its de-oligomerization resulting in the appearance of new monomeric band, it is clear that FnD affects the expression of P2Y12R isoforms. It has been shown that P2Y12R is essential in microglial sensing of ATP and ADP derived from neuronal activity (Badimon et al., 2020). Microglial processes are equipped with NTPDase1 and CD73, ectoenzymes responsible for ATP and ADP conversion to adenosine which acts upon A1 receptor on neuron membranes attenuating the neuronal overactivation, thus modulating their activity in health and disease (Badimon et al., 2020). Cserep et al. demonstrated that in ischemic injury in striatum microglia responds to changes in neuronal activity by increasing the process coverage of neurons in P2Y12R dependent manner, since inhibition of P2Y12R led to smaller process coverage, altered functional connectivity, increase of the injury area and worsened overall neurological outcome (Cserép et al., 2020). Taken together, changes observed regarding the increased intensity of precursor bands of β1 integrin and appearance of monomeric bands of P2Y12R imply that FnD affects their functional state, cellular localization and glycosylation levels though further studies are needed to elucidate the underlying mechanisms.

FnD treatment exhibited spatiotemporally distinct effects on microglia and astrocytes in the lesion in our current study. In injury microglial cells enter the wound area prior to astrocytes and this was observed in our control groups. The absence of TnC even further potentiated this effect as both density and proliferation of microglia were elevated, whereas the addition of FnD strongly diminished both the density and proliferation of microglia while promoting their activated phenotype. On the other hand, FnD induced migration of the astrocytes into the gap region, but did not affect their proliferation rates. Mander et al. observed that proliferation of rat microglia *in vitro* seemed to be regulated by hydrogen peroxide produced by NADPH oxidase upon stimulation with IL-1β and TNF-α, since it was completely abolished with the addition of NADPH oxidase inhibitors while this did not affect astrocyte proliferation (Mander et al., 2006). Additionally, it was shown that astrocytes and microglia respond differentially in a model of induced inflammation. While IL-6, PGE2, and cyclooxygenase 2 (COX2) release increased in astrocytes, completely different pro-inflammatory profile was observed in microglia with increased release of IL-1β, TNF-α, nitric oxide (NO), iNOS and STAT3 activation (Lu et al., 2014). Even though both cell types express integrins and TLR4, time of their activation and downstream signalling pathways may differ, since it has been shown that astrocytes and microglia have cell-specific Ca^2+^ signalling dynamics (Rieder et al., 2022). Astrocytes and microglia express multiple P2Y and P2X receptors but those receptors are differentially activated by nucleotides in a context specific manner upon pathophysiological conditions and further mediate different long-term changes of these cells during inflammatory gliosis (Abbracchio and Ceruti, 2006).

Finally, the time and place of TnC secretion are vital to its roles. It was shown in epithelial wound that fibrin-fibronectin provisional matrix is first deposited upon injury where fibroblasts are anchored via formation of actin stress fibers and as injury is being repaired, the matrix is being contracted. TnC is however transiently upregulated in boundary regions where it inhibits formation of actin stress fibres and focal adhesion through inhibition of FAK and RhoA activity, thus promoting motile phenotype and preventing premature matrix contraction (Midwood and Schwarzbauer, 2002). In our model FnD was exogenously applied. However, *in vivo* FnD produced by proteolytic cleavage by MMPs would be confined to specific regions mainly in the injury border zone where astrocytes are located. It is therefore plausible that in the border zone FnD inhibits excessive proliferation of microglia and promotes motile phenotype of astrocytes to functionally separate the injury region from the surrounding tissue. These findings could be even more complex if examined in multicellular models involving other cell types. Our results are offering a new view of the importance of TnC`s multiple roles and, further experiments involving 3D and organotypic culture models as well as *in vivo* experiments are needed to position this effect precisely in the sequence of events after injury.

## Data Availability

The raw data supporting the conclusion of this article will be made available by the authors, without undue reservation.

## References

[B1] Abbracchio M. P. Ceruti S. (2006). Roles of P2 receptors in glial cells: Focus on astrocytes. Purinergic Signal. 2, 595–604. 10.1007/s11302-006-9016-0 18404462 PMC2096663

[B2] Ahuja C. S. Wilson J. R. Nori S. Kotter M. R. N. Druschel C. Curt A. (2017). Traumatic spinal cord injury. Nat. Rev. Dis. Prim. 3, 17018. 10.1038/nrdp.2017.18 28447605

[B3] Badimon A. Strasburger H. J. Ayata P. Chen X. Nair A. Ikegami A. (2020). Negative feedback control of neuronal activity by microglia. Nature 586, 417–423. 10.1038/s41586-020-2777-8 32999463 PMC7577179

[B4] Bartsch S. Bartsch U. Dijrries U. Faissner A. Weller A. Ekblom P. (1992). Expression of tenascin in the developing and adult cerebellar cortex. J. Neurosci. 12 (3), 736–749. 10.1523/JNEUROSCI.12-03-00736.1992 1372043 PMC6576029

[B5] Beck K. D. Nguyen H. X. Galvan M. D. Salazar D. L. Woodruff T. M. Anderson A. J. (2010). Quantitative analysis of cellular inflammation after traumatic spinal cord injury: Evidence for a multiphasic inflammatory response in the acute to chronic environment. Brain 133, 433–447. 10.1093/brain/awp322 20085927 PMC2858013

[B6] Bellver-Landete V. Bretheau F. Mailhot B. Vallières N. Lessard M. Janelle M. E. (2019). Microglia are an essential component of the neuroprotective scar that forms after spinal cord injury. Nat. Commun. 10, 518. 10.1038/s41467-019-08446-0 30705270 PMC6355913

[B7] Bernier L. P. Bohlen C. J. York E. M. Choi H. B. Kamyabi A. Dissing-Olesen L. (2019). Nanoscale surveillance of the brain by microglia via cAMP-regulated filopodia. Cell Rep. 27, 2895–2908. e4. 10.1016/j.celrep.2019.05.010 31167136

[B8] Bijelić D. Adžić M. Perić M. Jakovčevski I. Förster E. Schachner M. (2021). Different functions of recombinantly expressed domains of tenascin-C in glial scar formation. Front. Immunol. 11, 624612. 10.3389/fimmu.2020.624612 33679718 PMC7934619

[B9] Bradbury E. J. Burnside E. R. (2019). Moving beyond the glial scar for spinal cord repair. Nat. Commun. 10, 3879. 10.1038/s41467-019-11707-7 31462640 PMC6713740

[B10] Broz P. Dixit V. M. (2016). Inflammasomes: Mechanism of assembly, regulation and signalling. Nat. Rev. Immunol. 16, 407–420. 10.1038/nri.2016.58 27291964

[B11] Cavanagh J. B. (1970). The proliferation of astrocytes around a needle wound in the rat brain. J. Anat. 106 (3), 471–487. 4912665 PMC1233423

[B12] Chen J. Joon Lee H. Jakovcevski I. Shah R. Bhagat N. Loers G. (2010). The extracellular matrix glycoprotein tenascin-c is beneficial for spinal cord regeneration. Mol. Ther. 18, 1769–1777. 10.1038/mt.2010.133 20606643 PMC2951554

[B13] Cherry J. D. Olschowka J. A. O’Banion M. K. (2014). Neuroinflammation and M2 microglia: The good, the bad, and the inflamed. J. Neuroinflammation 11, 98. 10.1186/1742-2094-11-98 24889886 PMC4060849

[B14] Chiquet-Ehrismann R. (1991). Anti-adhesive molecules of the extracellular matrix. Curr. Opin. Cell Biol. 3 (5), 800–804. 10.1016/0955-0674(91)90053-2 1718339

[B15] Chung C. Y. Erickson H. P. (1994). Cell surface annexin II is a high affinity receptor for the alternatively spliced segment of tenascin-C. J. Cell Biol. 126 (2), 539–548. 10.1083/jcb.126.2.539 7518469 PMC2200039

[B16] Chung C. Y. Murphy-Ullrich J. E. Erickson H. P. (1996). Mitogenesis, cell migration, and loss of focal adhesions induced by tenascin-C interacting with its cell surface receptor, annexin II. Mol. Biol. Cell 7 (6)–88392. 10.1091/mbc.7.6.883 PMC275940 8816995

[B17] Crossin K. L. (1991). Cytotactin binding: Inhibition of stimulated proliferation and intracellular alkalinization in fibroblasts. Proc. Natl. Acad. Sci. U. S. A. 88 (24), 11403–11407. (morphogene^sl/growth control/second messengersx/telilar matrix). 10.1073/pnas.88.24.11403 1722330 PMC53143

[B19] Cserép C. Pósfai B. Lénárt N. Fekete R. László Z. I. Lele Z. (2020). Microglia monitor and protect neuronal function through specialized somatic purinergic junctions. Science 367, 528–537. 10.1126/science.aax6752 31831638

[B20] Davalos D. Grutzendler J. Yang G. Kim J. v. Zuo Y. Jung S. (2005). ATP mediates rapid microglial response to local brain injury *in vivo* . Nat. Neurosci. 8, 752–758. 10.1038/nn1472 15895084

[B21] Davis E. J. Foster T. D. ∼0∼s’ W. E. Cetluiur W. E. T. (1994). Cellular forms and functions of brain microglia. Brain Res. Bull. 34 (1), 73–78. 10.1016/0361-9230(94)90189-9 8193937

[B22] Deng S. Yu K. Zhang B. Yao Y. Wang Z. Zhang J. (2015). Toll-like receptor 4 promotes NO synthesis by upregulating GCHI expression under oxidative stress conditions in sheep monocytes/macrophages. Oxid. Med. Cell. Longev. 2015, 359315. 10.1155/2015/359315 26576220 PMC4630417

[B23] Dorries U. Taylor J. Xiao Z. Lochter A. Montag D. Schachner M. (1996). Distinct effects of recombinant tenascin-C domains on neuronal cell adhesion, growth cone guidance, and neuronal polarity. J. Neurosci. Res. 43 (4), 420–438. 10.1002/(SICI)1097-4547(19960215)43:4<420::AID-JNR4>3.0.CO;2-H 8699529

[B24] Erickson H. P. (1993). Tenascin-C, tenascin-R and tenascin-X: A family of talented proteins in search of functions. Curr. Opin. Cell Biol. 5 (5), 869–876. 10.1016/0955-0674(93)90037-q 7694605

[B25] Escartin C. Galea E. Lakatos A. O’Callaghan J. P. Petzold G. C. Serrano-Pozo A. (2021). Reactive astrocyte nomenclature, definitions, and future directions. Nat. Neurosci. 24, 312–325. 10.1038/s41593-020-00783-4 33589835 PMC8007081

[B26] Evers M. R. Salmen B. Bukalo O. Rollenhagen A. Bö Sl M. R. Morellini F. (2002). Impairment of L-type Ca 2 channel-dependent forms of hippocampal synaptic plasticity in mice deficient in the extracellular matrix glycoprotein tenascin-C. J. Neurosci. 22 (16), 7177–7194. 10.1523/JNEUROSCI.22-16-07177.2002 12177213 PMC6757873

[B27] Faissner A. Steindler D. (1995). Boundaries and inhibitory molecules in developing neural tissues. Glia 13 (4), 233–254. 10.1002/glia.440130402 7615335

[B28] Faissner A. (1997). The tenascin gene family in axon growth and guidance. Springer-Verlag. 10.1007/s004410050938 9321695

[B29] Fernández-Arjona M. del M. Grondona J. M. Granados-Durán P. Fernández-Llebrez P. López-Ávalos M. D. (2017). Microglia morphological categorization in a rat model of neuroinflammation by hierarchical cluster and principal components analysis. Front. Cell. Neurosci. 11, 235. 10.3389/fncel.2017.00235 28848398 PMC5550745

[B30] Giblin S. P. Midwood K. S. (2015). Tenascin-C: Form versus function. Cell adh. Migr. 9, 48–82. 10.4161/19336918.2014.987587 25482829 PMC4422809

[B31] Giordana M. T. Attanasio A. Cavalla P. Migheli A. Vigliani M. C. Schiffer D. (1994). Reactive cell proliferation and microglia following injury t o the rat brain. Neuropathol. Appl. Neurobiol. 20 (2), 163–174. 10.1111/j.1365-2990.1994.tb01175.x 8072646

[B32] Goshi N. Morgan R. K. Lein P. J. Seker E. (2020). A primary neural cell culture model to study neuron, astrocyte, and microglia interactions in neuroinflammation. J. Neuroinflammation 17, 155. 10.1186/s12974-020-01819-z 32393376 PMC7216677

[B33] Gotz B. Scholze A. Clement A. Joester A. Schiitte K. Wigger F. (1996). Tenascin-C contains distinct adhesive, anti-adhesive, and neurite outgrowth promoting sites for neurons. J. Cell Biol. 132 (4), 681–699. 10.1083/jcb.132.4.681 8647898 PMC2199878

[B35] Gulcher J. R. Nies D. E. Marton L. S. Stefansson K. (1989). An alternatively spliced region of the human hexabrachion contains a repeat of potential N-glycosylation sites (type III homology units/epidermal growth factor motifs/fibrinogen/primary structure). Proc. Natl. Acad. Sci. U. S. A. 86 (5), 1588–1592. 10.1073/pnas.86.5.1588 2466295 PMC286743

[B36] Haage V. Elmadany N. Roll L. Faissner A. Gutmann D. H. Semtner M. (2019). Tenascin C regulates multiple microglial functions involving TLR4 signaling and HDAC1. Brain Behav. Immun. 81, 470–483. 10.1016/j.bbi.2019.06.047 31271872

[B37] Hang Q. Isaji T. Hou S. Wang Y. Fukuda T. Gu J. (2017). A key regulator of cell adhesion: Identification and characterization of important N -glycosylation sites on integrin α5 for cell migration. Mol. Cell. Biol. 37, e00558-16. 10.1128/mcb.00558-16 28167607 PMC5394273

[B38] Haynes S. E. Hollopeter G. Yang G. Kurpius D. Dailey M. E. Gan W. B. (2006). The P2Y12 receptor regulates microglial activation by extracellular nucleotides. Nat. Neurosci. 9, 1512–1519. 10.1038/nn1805 17115040

[B40] Isaji T. Sato Y. Fukuda T. Gu J. (2009). N-Glycosylation of the I-like domain of beta1 integrin is essential for beta1 integrin expression and biological function: identification of the minimal N-glycosylation requirement for alpha5beta1. J. Biol. Chem. 284, 12207–12216. 10.1074/jbc.M807920200 19261610 PMC2673289

[B41] Jakovcevski I. Miljkovic D. Schachner M. Andjus P. R. (2013). Tenascins and inflammation in disorders of the nervous system. Amino Acids 44, 1115–1127. 10.1007/s00726-012-1446-0 23269478

[B42] Jones F. S. Jones P. L. (2000). The tenascin family of ECM glycoproteins: Structure, function, and regulation during embryonic development and tissue remodeling. Dev. Dyn. 218 (2), 235–259. REVIEWS A PEER REVIEWED FORUM. 10.1002/(SICI)1097-0177(200006)218:2<235::AID-DVDY2>3.0.CO;2-G 10842355

[B43] Karperien A. L. (2013). FracLac for ImageJ. 10.13140/2.1.4775.8402

[B44] Karve I. P. Taylor J. M. Crack P. J. (2016). The contribution of astrocytes and microglia to traumatic brain injury. Br. J. Pharmacol. 173, 692–702. 10.1111/bph.13125 25752446 PMC4742296

[B45] Latov N. Nilaver G. Zimmerman E. A. Johnson W. G. Silverman A.-J. Defendini R. (1979). Fibrillary astrocytes proliferate in response to brain injury A study combining lmmunoperoxidase technique for glial fibrillary acidic protein and radioautography of tritiated thymidine. Dev. Biol. 72 (2), 381–384. 10.1016/0012-1606(79)90127-1 389711

[B46] Laywell E. D. Dorriest U. Bartscht U. Faissnert A. Schachnert M. Steindler D. A. (1992). Enhanced expression of the developmentally regulated extracellular matrix molecule tenascin following adult brain injury. Proc. Natl. Acad. Sci. U. S. A. 89 (7), 2634–2638. brain wounds/astrocytes/regeneration/*in situ* hybridization/immunocytochemistry. 10.1073/pnas.89.7.2634 1372985 PMC48716

[B47] Liddelow S. A. Guttenplan K. A. Clarke L. E. Bennett F. C. Bohlen C. J. Schirmer L. (2017). Neurotoxic reactive astrocytes are induced by activated microglia. Nature 541, 481–487. 10.1038/nature21029 28099414 PMC5404890

[B48] Liddelow S. A. Marsh S. E. Stevens B. (2020). Microglia and astrocytes in disease: Dynamic duo or partners in crime? Trends Immunol. 41, 820–835. 10.1016/j.it.2020.07.006 32819809

[B50] Lu Y. He M. Zhang Y. Xu S. Zhang L. He Y. (2014). Differential pro-inflammatory responses of astrocytes and microglia involve STAT3 activation in response to 1800 MHz radiofrequency fields. PLoS ONE 9, e108318. 10.1371/journal.pone.0108318 25275372 PMC4183530

[B51] Mander P. K. Jekabsone A. Brown G. C. (2006). Microglia proliferation is regulated by hydrogen peroxide from NADPH oxidase. J. Immunol. 176, 1046–1052. 10.4049/jimmunol.176.2.1046 16393992

[B52] Marzeda A. M. Midwood K. S. (2018). Internal affairs: Tenascin-C as a clinically relevant, endogenous driver of innate immunity. J. Histochem. Cytochem. 66, 289–304. 10.1369/0022155418757443 29385356 PMC5958381

[B53] Midwood K. Sacre S. Piccinini A. M. Inglis J. Trebaul A. Chan E. (2009). Tenascin-C is an endogenous activator of Toll-like receptor 4 that is essential for maintaining inflammation in arthritic joint disease. Nat. Med. 15, 774–780. 10.1038/nm.1987 19561617

[B54] Midwood K. S. Chiquet M. Tucker R. P. Orend G. (2016). Tenascin-C at a glance. J. Cell Sci. 129, 4321–4327. 10.1242/jcs.190546 27875272

[B55] Midwood K. S. Orend G. (2009). The role of tenascin-C in tissue injury and tumorigenesis. J. Cell Commun. Signal. 3, 287–310. 10.1007/s12079-009-0075-1 19838819 PMC2778592

[B56] Midwood K. S. Schwarzbauer J. E. (2002). Tenascin-C modulates matrix contraction via focal adhesion kinase- and Rho-mediated signaling pathways. Mol. Biol. Cell 13, 3601–3613. 10.1091/mbc.E02-05-0292 12388760 PMC129969

[B57] Moreno-Layseca P. Icha J. Hamidi H. Ivaska J. (2019). Integrin trafficking in cells and tissues. Nat. Cell Biol. 21, 122–132. 10.1038/s41556-018-0223-z 30602723 PMC6597357

[B58] Morgan M. R. Hamidi H. Bass M. D. Warwood S. Ballestrem C. Humphries M. J. (2013). Syndecan-4 phosphorylation is a control point for integrin recycling. Dev. Cell 24, 472–485. 10.1016/j.devcel.2013.01.027 23453597 PMC3605578

[B59] Morrison H. Young K. Qureshi M. Rowe R. K. Lifshitz J. (2017). Quantitative microglia analyses reveal diverse morphologic responses in the rat cortex after diffuse brain injury. Sci. Rep. 7, 13211. 10.1038/s41598-017-13581-z 29038483 PMC5643511

[B91] Murphy-Ullrich J. E. Lightner V. A. Aukhil I. Yan Y. Z. Erickson H. P. Höök M. (1991). Focal adhesion integrity is downregulated by the alternatively spliced domain of human tenascin. J. Cell. Biol. 116 (3), 833. 10.1083/jcb.115.4.1127 PMC2289958 1720121

[B60] Nasu-Tada K. Koizumi S. Inoue K. (2005). Involvement of beta1 integrin in microglial chemotaxis and proliferation on fibronectin: different regulations by ADP through PKA. GLIA 52, 98–107. 10.1002/glia.20224 15920726

[B61] Nies D. E. Hemesath T. J. Kim J. H. Gulcher J. R. Stefansson K. (1991). The complete cDNA sequence of human hexabrachion (tenascin): A multidomain protein containing unique epidermal growth factor repeats. J. Biol. Chem. 266, 2818–2823. 10.1016/s0021-9258(18)49920-6 1704365

[B62] Nimmerjahn A. Kirchhoff F. Helmchen F. (2005). Resting microglial cells are highly dynamic surveillants of brain parenchyma *in vivo* . Science 308, 1314–1318. 10.1126/science.1110647 15831717

[B63] Nishio T. Kawaguchi S. Iseda T. Kawasaki T. Hase T. (2003). Secretion of tenascin-C by cultured astrocytes: Regulation of cell proliferation and process elongation. Brain Res. 990, 129–140. 10.1016/S0006-8993(03)03448-6 14568337

[B64] Nishio T. Kawaguchi S. Yamamoto M. Iseda T. Kawasaki T. Hase T. (2005). Tenascin-C regulates proliferation and migration of cultured astrocytes in a scratch wound assay. Neuroscience 132, 87–102. 10.1016/j.neuroscience.2004.12.028 15780469

[B65] Okada T. Suzuki H. (2021). The role of tenascin-C in tissue injury and repair after stroke. Front. Immunol. 11, 607587. 10.3389/fimmu.2020.607587 33552066 PMC7859104

[B66] Orend G. Huang W. Olayioye M. A. Hynes N. E. Chiquet-Ehrismann R. (2003). Tenascin-C blocks cell-cycle progression of anchorage-dependent fibroblasts on fibronectin through inhibition of syndecan-4. Oncogene 22, 3917–3926. 10.1038/sj.onc.1206618 12813465

[B67] Orihuela R. McPherson C. A. Harry G. J. (2016). Microglial M1/M2 polarization and metabolic states. Br. J. Pharmacol. 173, 649–665. 10.1111/bph.13139 25800044 PMC4742299

[B68] Prieto A. L. Andersson-Fisone C. Crossin K. L. (1992). Characterization of multiple adhesive and counteradhesive domains in the extracellular matrix protein cytotactin. J. Cell Biol. 119 (3), 663–678. 10.1083/jcb.119.3.663 1383239 PMC2289676

[B69] Rankin C. R. Hilgarth R. S. Leoni G. Kwon M. den Beste K. A. Parkos C. A. (2013). Annexin A2 regulates β1 integrin internalization and intestinal epithelial cell migration. J. Biol. Chem. 288, 15229–15239. 10.1074/jbc.M112.440909 23558678 PMC3663542

[B70] Rhodes K. E. Moon L. D. F. Fawcett J. W. (2003). Inhibiting cell proliferation during formation of the glial scar: Effects on axon regeneration in the CNS. Neuroscience 120, 41–56. 10.1016/S0306-4522(03)00285-9 12849739

[B71] Rieder P. Gobbo D. Stopper G. Welle A. Damo E. Kirchhoff F. (2022). Astrocytes and microglia exhibit cell-specific Ca2+ signaling dynamics in the murine spinal cord. Front. Mol. Neurosci. 15, 840948. 10.3389/fnmol.2022.840948 35431801 PMC9006623

[B72] Saito Y. Imazeki H. Miura S. Yoshimura T. Okutsu H. Harada Y. (2007). A peptide derived from tenascin-C induces beta1 integrin activation through syndecan-4. J. Biol. Chem. 282, 34929–34937. 10.1074/jbc.M705608200 17901052

[B73] Savi P. Zachayus J.-L. Delesque-Touchard N. Labouret C. Herve C. Uzabiaga M.-F. (2006). The active metabolite of Clopidogrel disrupts P2Y12 receptor oligomers and partitions them out of lipid rafts. Proc. Natl. Acad. Sci. U. S. A. 103, 11069–11074. 10.1073/pnas.0510446103 16835302 PMC1635153

[B74] Scholze A. Gotz B. Faissner A. (1996). Glial cell interactionswith tenascin-C: Adhesion and repulsionto different tenascin-C domains is cell type related. Int. J. Dev. Neurosci. 14315 (3)–29. 10.1016/0736-5748(96)00016-0 8842807

[B75] Seiffert M. I. Beck S. C. Schermutzki F. Muller C. A. Erickson H. P. Klein G. (1998). Mitogenic and adhesive effects of tenascin-C on human hematopoietic CelLs are mediated by various functional domains. Matrix Biol. 17 (1), 47–63. 10.1016/s0945-053x(98)90124-x 9628252

[B76] She S. Xu B. He M. Lan X. Wang Q. (2010). Open Access RESEARCH Nm23-H1 suppresses hepatocarcinoma cell adhesion and migration on fibronectin by modulating glycosylation of integrin beta1. Available at: http://www.jeccr.com/content/29/1/93 . 10.1186/1756-9966-29-93 PMC2909969 20618991

[B77] Slovinska L. Blasko J. Nagyova M. Szekiova E. Cizkova D. (2016). “ *In vitro* models of spinal cord injury,” in Recovery of motor function following spinal cord injury. Editor Fuller H. Gates M. (London: InTech). 10.5772/63459

[B78] Sriramarao P. Mendler M. Bourdon M. A. (1993). Endothelial cell attachment and spreading on human tenascin is mediated by α2β1 and αvβ3 integrins. J. Cell Sci. 105, 1001–1012. 10.1242/jcs.105.4.1001 7693733

[B79] Susarla B. T. S. Villapol S. Yi J. H. Geller H. M. Symes A. J. (2014). Temporal patterns of cortical proliferation of glial cell populations after traumatic brain injury in mice. ASN Neuro 6, 159–170. 10.1042/AN20130034 24670035 PMC4013687

[B80] Taylor H. C. Lightner V. A. Beyer W. F. Mccaslin D. Briscoe G. Erickson H. P. (1989). Biochemical and structural studies of tenascin/hexabrachion proteins. J. Cell Biochem. 41 (2), 71–90. 10.1002/jcb.240410204 2482292

[B81] Tucker R. P. Chiquet-Ehrismann R. (2015). Tenascin-C: Its functions as an integrin ligand. Int. J. Biochem. Cell Biol. 65, 165–168. 10.1016/j.biocel.2015.06.003 26055518

[B82] Tucker R. P. Chiquet-Ehrismann R. (2009). The regulation of tenascin expression by tissue microenvironments. Biochim. Biophys. Acta 1793, 888–892. 10.1016/j.bbamcr.2008.12.012 19162090

[B83] Walker F. R. Beynon S. B. Jones K. A. Zhao Z. Kongsui R. Cairns M. (2014). Dynamic structural remodelling of microglia in health and disease: A review of the models, the signals and the mechanisms. Brain Behav. Immun. 37, 1–14. 10.1016/j.bbi.2013.12.010 24412599

[B84] Werner C. Engelhard K. (2007). Pathophysiology of traumatic brain injury. Br. J. Anaesth. 99, 4–9. 10.1093/bja/aem131 17573392

[B85] Wiese S. Karus M. Faissner A. (2012). Astrocytes as a source for extracellular matrix molecules and cytokines. Front. Pharmacol. 3, 120. 10.3389/fphar.2012.00120 22740833 PMC3382726

[B86] Yoo J. Y. Hwang C. H. Hong H. N. (2016). A model of glial scarring analogous to the environment of a traumatically injured spinal cord using kainate. Ann. Rehabil. Med. 40, 757–768. 10.5535/arm.2016.40.5.757 27847705 PMC5108702

[B87] Yoshida T. Akatsuka T. Imanaka-Yoshida K. (2015). Tenascin-C and integrins in cancer. Cell adh. Migr. 9, 96–104. 10.1080/19336918.2015.1008332 25793576 PMC4422796

[B88] Zhang Y. Peti-Peterdi J. Müller C. E. Carlson N. G. Baqi Y. Strasburg D. L. (2015). P2Y12 receptor localizes in the renal collecting duct and its blockade augments arginine vasopressin action and alleviates nephrogenic diabetes insipidus. J. Am. Soc. Nephrol. 26, 2978–2987. 10.1681/ASN.2014010118 25855780 PMC4657822

[B89] Zhou X. Wahane S. Friedl M. S. Kluge M. Friedel C. C. Avrampou K. (2020). Microglia and macrophages promote corralling, wound compaction and recovery after spinal cord injury via Plexin-B2. Nat. Neurosci. 23, 337–350. 10.1038/s41593-020-0597-7 32112058 PMC7412870

[B90] Zuliani-Alvarez L. Marzeda A. M. Deligne C. Schwenzer A. McCann F. E. Marsden B. D. (2017). Mapping tenascin-C interaction with toll-like receptor 4 reveals a new subset of endogenous inflammatory triggers. Nat. Commun. 8, 1595. 10.1038/s41467-017-01718-7 29150600 PMC5693923

