# Supplementary material for: Tenascin-C fibronectin D domain is involved in the fine-tuning of glial response to CNS injury in vitro
Source: Front Cell Dev Biol. 2022 Aug 26;10:952208. doi: 10.3389/fcell.2022.952208 (PMC9462431; doi:10.3389/fcell.2022.952208)
Supplement: Supplementary file 1 [file DataSheet1.pdf]

Supplementary material

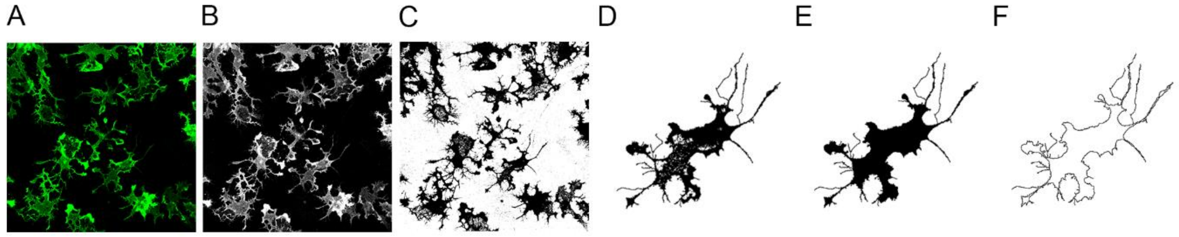

Supplementary Figure 1. Image pre-processing for morphometric analysis. RGB images of microglia in the gap area (A) were transformed into 8-bit grayscale (B) and binarized (C). Images were then manually edited to obtain continuous set of pixels for chosen cell that was then cropped (D), and filled (E) or outlined (F) shape was used for further analysis.

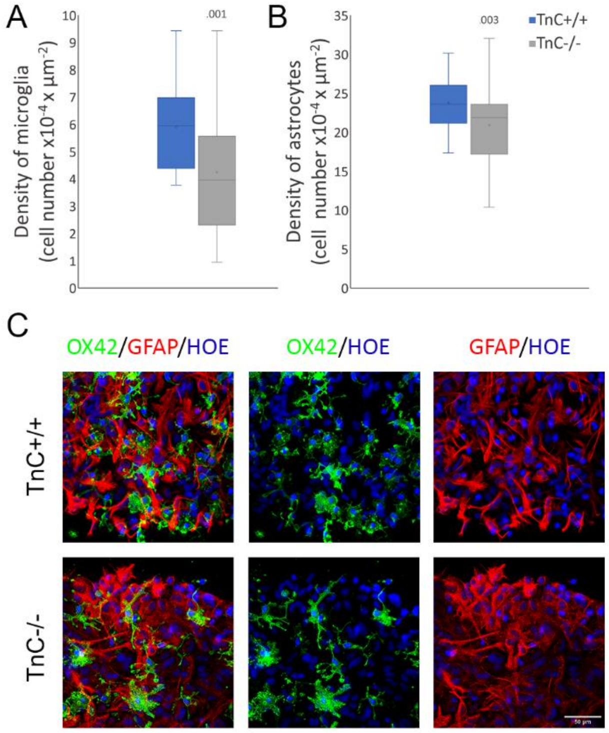

Supplementary Figure 2. Initial densities of microglia and astrocytes are higher in TnC+/+ than TnC-/- cultures. Box-whisker plots of densities of microglia (A) and astrocytes (B) in mixed cultures. Independent-samples t-test was done, p values indicated. Representative confocal micrographs of immunofluorescently labelled microglia (OX-42, green), astrocytes (GFAP, red) and nuclei labelled with Hoechst (HOE, blue) are shown for both genotypes (C). Sale bar indicated: 50µm. (n=3 cultures)

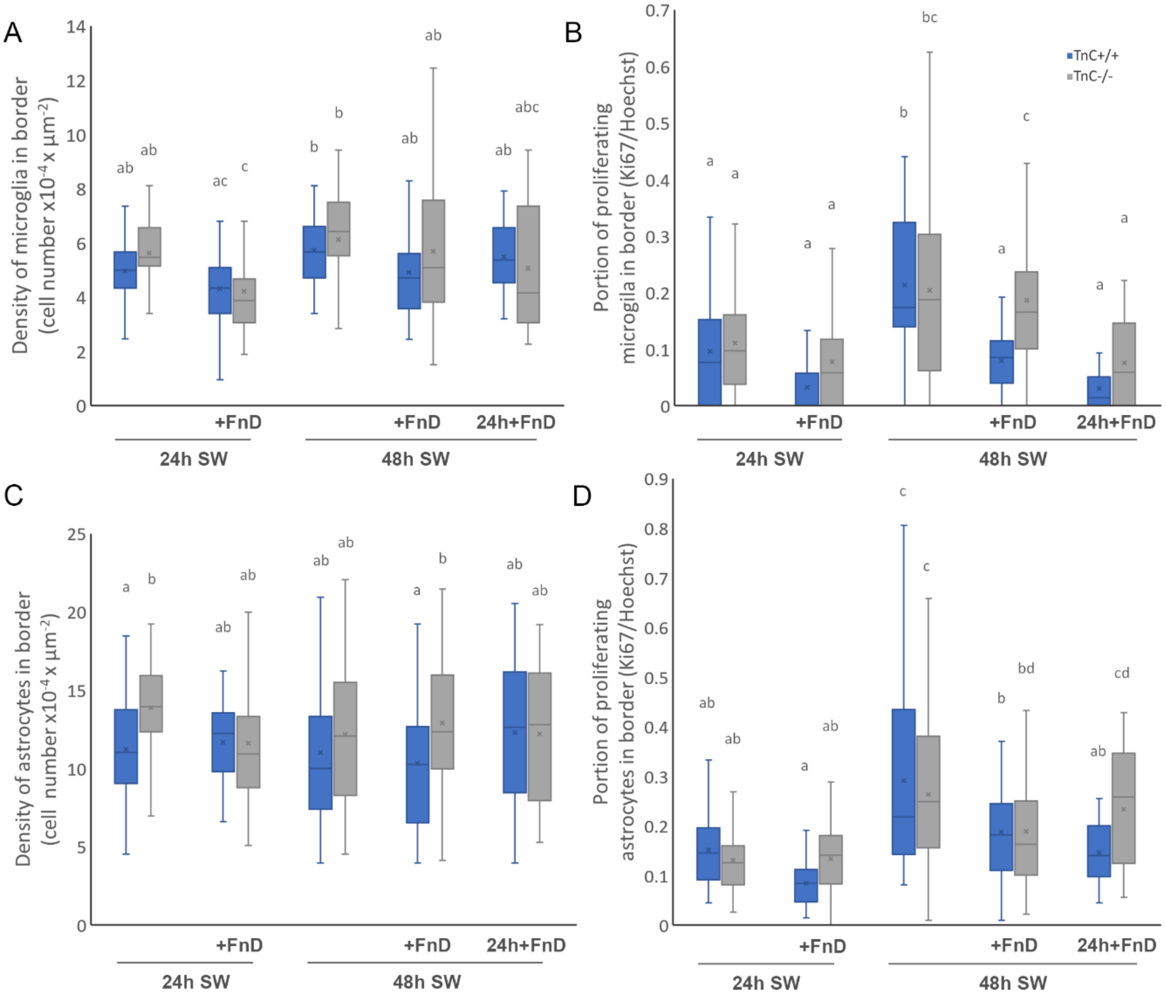

Supplementary Figure 3. FnD treatment lowers microglial density in the first 24h upon SW, and decreases proliferation of both microglia and astrocytes 48h upon SW in the border region. Confocal images of cells labelled with immunofluorescent markers for microglia, astrocytes, cell nuclei and proliferation, were used for calculations of density and proliferation rates. Box – whisker plots show densities and proliferation rates of microglia (A, B, resp.) and astrocytes (C, D, resp.) in the border region. Two-way ANOVA was used to test statistical significance (*p<.05*). Pairwise comparisons are denoted with letters, shared letters imply no significant difference between groups. (No. images=36, from 3 independent cultures, per treatment, per genotype)

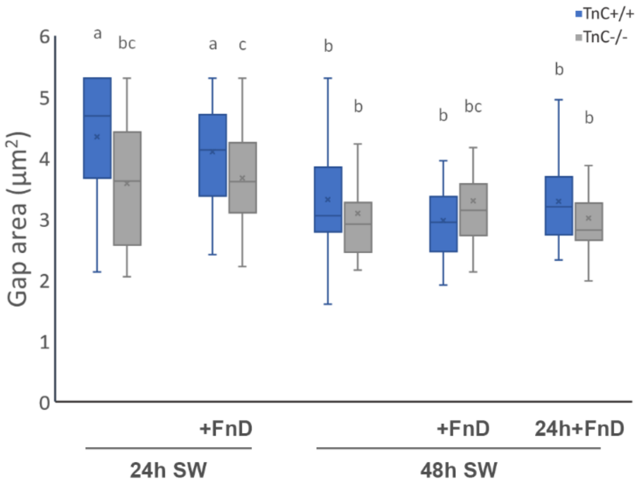

Supplementary Figure 4. Gap areas are smaller in the absence of TnC in the first 24h upon SW, while FnD treatment has no effect. Box-whisker plots of gap areas are shown. Two-way ANOVA was used to test statistical significance ($p<.05$). Pairwise comparisons are denoted with letters, shared letters imply no significant difference between groups. (n=3 cultures)

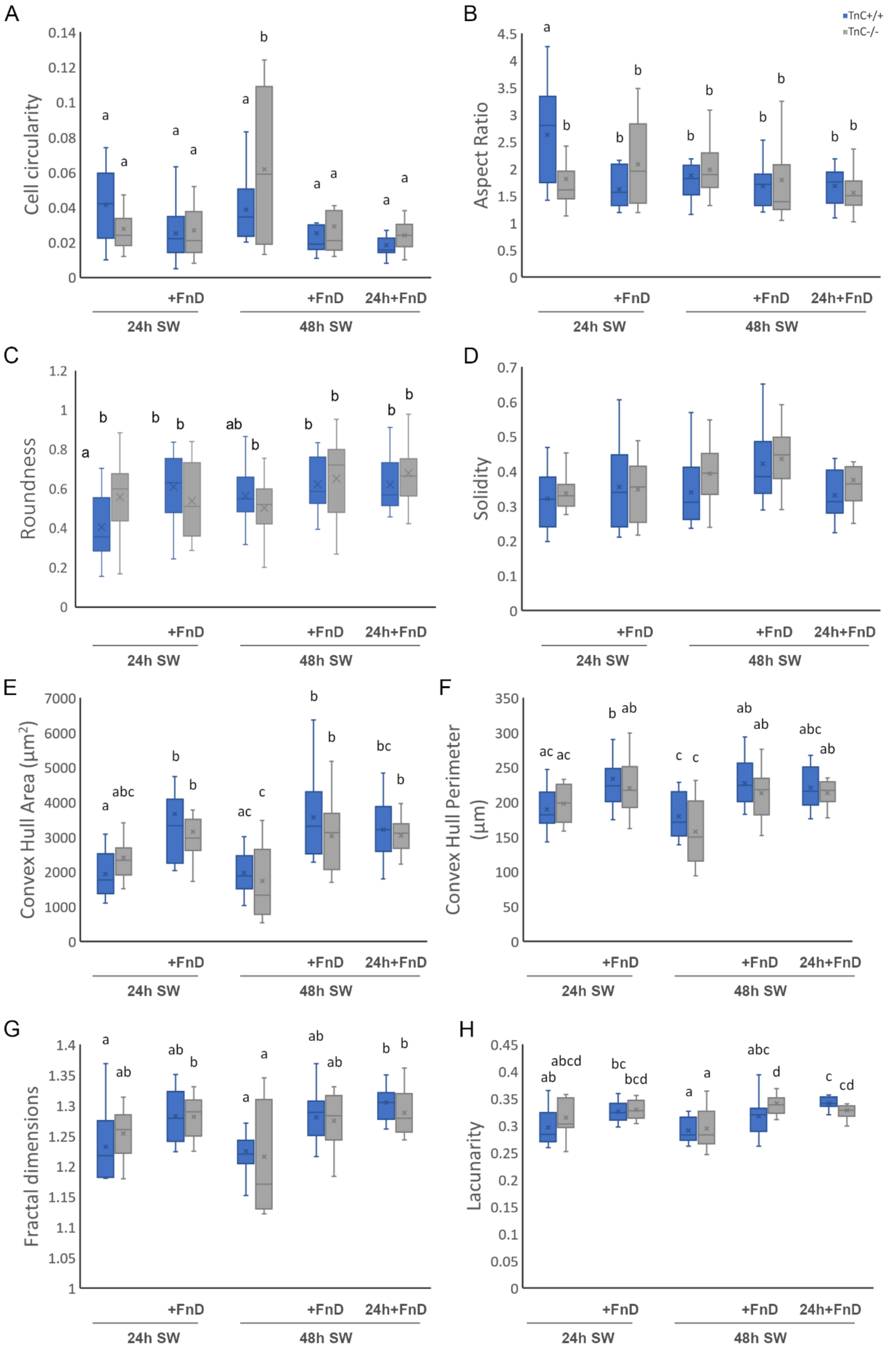
A
B
C
D
E
F
G
H
TnC+/+
TnC-/-
Cell circularity
Aspect Ratio
Roundness
Solidity
Convex Hull Area (µm²)
Convex Hull Perimeter (µm)
Fractal dimensions
Lacunarity
+FnD
24h+FnD
24h SW
48h SW

Supplementary Figure 5. Additional morphometric parameters of microglial cells in the gap area. Box – whisker plots of Cell circularity (A), Aspect Ratio (B), Roundness (C), Solidity (D), Convex Hull Area (E), Convex Hull Perimeter (F), Fractal dimensions (G), Lacunarity (H) are shown. Two-way ANOVA was used to test statistical significance (*p*<*.05*). Pairwise comparisons are denoted with letters, shared letters imply no significant difference between groups.

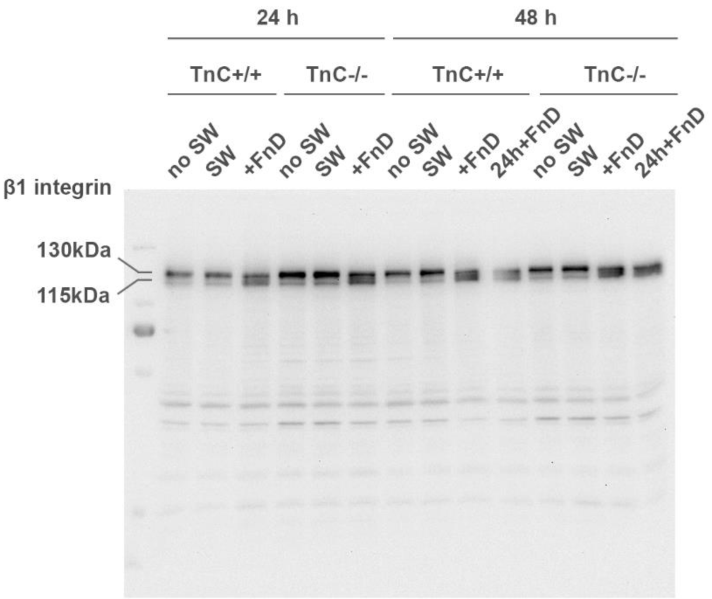

Supplementary Figure 6. Image of the whole Western blot membrane for β1 integrin (shown in Figure 7).
